# Sex-specific prediction models for aortic aneurysm integrating traditional clinical risk factors and proteomic profiles: a large-scale prospective study from the UK Biobank

**DOI:** 10.1186/s13293-026-00913-w

**Published:** 2026-05-11

**Authors:** Xuefei Han, Jiacheng Ding, Suyin Feng, Yunqian Li, Yiyin Gao, Runfeng Sun

**Affiliations:** 1 Donghai County People’s Hospital (Affiliated Kangda College of Nanjing Medical University), Lianyungang, 222000 China; 2 Department of Neurosurgery, The First Hospital of Jilin University, Changchun, 130021 China; 3 The Second Hospital of Jilin University, Changchun, 130041 China; 4 Cardio-Cerebral Vascular Disease Prevention and Treatment Innovation Center, Donghai County People’s Hospital (Affiliated Kangda College of Nanjing Medical University), Lianyungang, 222000 China; 5 Department of Endocrinology, The second Affiliated Hospital of Jilin University, Changchun, 130041 China

**Keywords:** Aortic aneurysm, Sex factors, Clinical risk factors, prospective analysis, Proteomic profiles

## Abstract

**Background:**

This study aimed to systematically map the sex-specific clinical and proteomic risk profiles of aortic aneurysm (AA), elucidate its molecular mechanisms, and develop a sex-specific protein risk prediction score.

**Methods:**

Based on the UK Biobank, we adopted a sex-stratified strategy to assess associations between traditional clinical factors and AA in 471,660 participants, and performed proteomics analysis on 49,887 participants with plasma protein data. Mediation analysis was used to explore the molecular mechanisms by which clinical risks drive AA. Finally, sex-specific protein risk scores were developed via LASSO regression, and their predictive performance was evaluated in an independent validation cohort.

**Results:**

Smoking (HR: Male: 2.82; Female: 4.42) and valvular disease (HR: Male: 2.01; Female: 4.62) were the strongest shared risk factors, with women exhibiting significantly higher susceptibility. Incident AA was primarily attributed to smoking (Male: 15.3%; Female: 19.8%) and hypertension (Male: 11.2%; Female: 10.2%). Smoking was associated with AA potentially through the ECM degradation pathway in both sexes. Hypertension may also influence AA risk through this pathway in men, whereas in women, it may primarily operate through metabolic and growth factor regulation pathways. Among LASSO-selected proteins, 4 were shared, 10 were male-specific, and 9 were female-specific. Ultimately, the model integrating traditional risk factors and sex-specific protein scores demonstrated superior predictive performance in an independent cohort (C-statistic: Male: 0.809; Female: 0.832).

**Conclusions:**

Smoking and hypertension are primary risk factors for AA. Men may be predisposed to structural destruction potentially mediated by ECM degradation, whereas women may be predisposed to intrinsic failure potentially involving metabolic dysregulation and cell apoptosis. The model integrating age, clinical factors, and protein scores better captures residual risk, significantly improving AA prediction.

**Supplementary Information:**

The online version contains supplementary material available at 10.1186/s13293-026-00913-w.

## Abstract (plain language summary)

Aortic aneurysm (AA) refers to a dangerous dilation of the aorta, the body’s largest blood vessel. It often presents no obvious symptoms before a rupture occurs, an event that carries an extremely high fatality rate. Although previous research has explored traditional clinical risk factors, the existence of significant sex differences has remained largely unexamined. Utilizing large-scale real-world data from nearly 500,000 individuals, this study provides an in-depth investigation into the impact of sex differences on AA. We found that smoking and valvular heart disease are the top risks shared by both sexes; however, women exhibit a far higher sensitivity to these risks than men.

Based on proteomics, the research reveals distinct biological mechanisms underlying the disease’s onset in men versus women: For men, smoking and hypertension primarily lead to the destruction of the vascular wall’s “structural scaffold” resulting in physical damage akin to “old pipes.” In contrast, for women, the disease is more frequently triggered by interferences with cellular repair and energy metabolism, resembling an “internal system failure.”

Accounting for these sex differences, we constructed unique protein risk scores for both males and females. The results demonstrate that among the 23 proteins associated with AA, only 4 are common to both sexes. Combining these protein scores with traditional risk factors enables a more accurate prediction of high-risk populations. This study serves as a critical reminder that cardiovascular health should prioritize sex differences. Furthermore, through such sex-specific blood tests, doctors may be able to identify high-risk individuals much earlier in the future, achieving true personalized prevention.

**Supplementary Information:**

The online version contains supplementary material available at 10.1186/s13293-026-00913-w.

## Highlights

- Smoking and valvular disease are the strongest shared risks for aortic aneurysm (AA) in both men and women, though women exhibit significantly higher susceptibility than men.
- Incident AA is primarily attributed to smoking and hypertension. Smoking drives the occurrence of AA through the extracellular matrix (ECM) degradation pathway in both sexes. While hypertension also drives AA via this same pathway in men, in women, it is primarily driven by metabolic and growth factor regulation pathways.
- Proteomic analysis identified 23 AA-associated proteins, with only 4 shared between the sexes—underscoring the necessity of sex-specific diagnostic approaches. Furthermore, a model integrating age, traditional clinical risk factors, and sex-specific protein scores better captures residual risk, achieving superior predictive performance (C-statistics: 0.809 for men; 0.832 for women).

**Supplementary Information:**

The online version contains supplementary material available at 10.1186/s13293-026-00913-w.

## Introduction

Aortic aneurysm (AA) is the second most common aortic disease and a leading cause of cardiovascular mortality worldwide. AA typically progresses insidiously; patients frequently remain asymptomatic until aneurysm rupture or aortic dissection (AD) occurs, both of which carry an extremely high mortality rate [1]. Globally, 150,000 to 200,000 deaths are attributed to AA rupture annually [2]. Current treatment options for AA are largely limited to open surgical repair and endovascular stent grafting, with no effective pharmacological therapies available [3–5]. Therefore, early identification of high-risk individuals is of considerable clinical importance for reducing the burden of AA-related morbidity and mortality.

Previous studies have established associations between traditional risk factors—including age, sex, smoking, hyperlipidemia, and hypertension—and the risk of incident AA [6–10]. For example, daily smokers have been shown to have a significantly higher risk of developing abdominal aortic aneurysm (AAA) compared with never-smokers [11, 12]. A study based on the ARIC cohort demonstrated that men have a significantly higher risk of developing AAA compared with women [13]. A meta-analysis encompassing over 5.4 million participants indicated that individuals with hypertension have an approximately 66% higher risk of developing AAA compared with those without hypertension [14]. Although these traditional risk factors collectively constitute the foundation of current clinical risk stratification strategies for AA, important knowledge gaps remain to be addressed. First, the underlying pathophysiological mechanisms linking these traditional clinical risk factors to AA remain unelucidated, creating barriers to understanding the complex relationships between these risk factors and the disease. Second, although traditional risk factors are major determinants of AA, relying solely on these variables has limited capacity to identify high-risk individuals, suggesting that integrating novel biomarkers may help to further improve risk stratification. Third, while the pathogenesis and risk profiles of AA may differ significantly between men and women, our understanding of the underlying sex-specific biological mechanisms remains limited.

Plasma proteomics offers a powerful complementary approach to address these knowledge gaps. By enabling simultaneous quantification of thousands of circulating proteins, proteomics can capture dynamic biological processes—such as extracellular matrix remodeling, inflammatory signaling, and metabolic dysregulation—that are not reflected by conventional clinical variables alone. Importantly, proteomic profiles may differ substantially between men and women, providing a unique opportunity to uncover sex-specific molecular mechanisms underlying AA and to identify novel biomarkers that improve risk stratification beyond traditional risk factors. Contemporary proteomics technologies, employing the Proximity Extension Assay (PEA) from the Olink platform, enable the concurrent analysis of thousands of proteins, thereby facilitating the longitudinal evaluation of protein-disease associations. Prior investigations have explored the relationships between specific plasma proteins, such as MMP12 and IL-6, and aortic aneurysm [15, 16]. However, most of these studies were limited by cross-sectional designs or restricted proteomic coverage. A recent study utilizing the UK Biobank identified protein biomarkers for a composite outcome of AA or aortic dissection (AD), confirming that the incorporation of these relevant proteins enhances predictive capability for AA or AD [17]. While undoubtedly valuable, that study was limited by phenotypic heterogeneity, as it did not distinguish between AA and AD. Furthermore, it merely adjusted for sex as a covariate rather than employing a sex-stratified analysis strategy. This heterogeneity in outcomes and the oversight of sex differences masked the unique pathophysiological characteristics of AA and hindered the development of precision prediction models. Currently, there is a lack of large-scale, prospective research to systematically map the sex-specific proteomic landscape of AA, elucidate the molecular pathways linking clinical risk factors to disease onset, and better identify populations at high risk for AA.

To address these knowledge gaps, we leveraged a large-scale, long-term prospective cohort to comprehensively characterize the sex-specific clinical and proteomic risk profiles of AA. Functional enrichment analysis was performed on proteins significantly associated with AA risk, followed by mediation analysis to quantify the proportion of the clinical risk–AA association mediated by specific proteomic pathways, thereby elucidating potential molecular mechanisms linking established risk factors to AA onset. Critically, all analyses were conducted with an explicit focus on sex-specific differences. As a proof-of-concept, sex-stratified protein risk scores were developed and validated for their association with incident AA risk. Finally, a predictive framework integrating traditional clinical risk factors and proteomic data was constructed separately for men and women, offering novel insights for the early identification of individuals at high risk for AA.

## Methods

### Study population

This prospective cohort study was conducted using data from the UK Biobank. The UK Biobank constitutes a large-scale longitudinal cohort that enrolled over 500,000 participants aged 40–69 years from 22 clinical assessment facilities across England, Scotland, and Wales from 2006 to 2010. The study gathered comprehensive phenotypic and genotypic data from participants via touchscreen surveys, clinical evaluations, and biospecimen analyses, with long-term follow-ups conducted. Details of the study design and data collection procedures for UKB are available online (https://www.ukbiobank.ac.uk). The study was approved by the UK Research Ethics Committee (reference: 11/NW/0382) and the Institutional Review Board of Tulane University (2018 − 1872). All individuals provided written informed consent [18, 19], and this research was conducted under UK Biobank application number 629,227. Initially, 501,935 participants were enrolled. We applied the following exclusion criteria at baseline: (i) a prior diagnosis of AA or AD (*n* = 584); (ii) congenital heart disease (*n* = 164); (iii) history of traumatic injury to the thoracic or abdominal aorta (*n* = 0); and (iv) missing data on covariates (*n* = 29,527). The final analysis for clinical risk factors included 471,660 participants.

### Outcome ascertainment

The primary outcome of this study was AA. The date and cause of death were obtained from death certificates held by the National Health Service (NHS) Information Centre (England and Wales) and the NHS Central Register Scotland (Scotland). Dates and causes of hospital admissions were identified via record linkage to Health Episode Statistics (England and Wales) and the Scottish Morbidity Records (SMR01) (Scotland). Data on disease diagnosis and follow-up were available until September 2023, and mortality data were available until July 2024. According to the International Classification of Diseases, Tenth Revision (ICD-10), AA was defined by the codes I71.1 through I71.9. TAA is defined as I71.1–I71.2, and AAA is defined as I71.3–I71.4. Supplementary Table 1 presents the corresponding ICD-10 codes used for the diagnosis of AA. Supplementary Table 2 presents the distribution of aortic aneurysm subtypes.

### Definitions of risk factors and covariates

This study evaluated eight common traditional clinical risk factors including socioeconomic status (SES), obesity, smoking, hypertension, hyperlipidemia, coronary heart disease, stroke, and valvular heart disease history. SES was dichotomized into high and low groups based on the median of the Townsend deprivation index (TDI). A higher TDI score indicates lower SES. According to World Health Organization (WHO) criteria, obesity was defined as a body mass index (BMI) ≥ 30.0 kg/m² [20]. Smoking status was categorized as never, former, or current smoker. Coronary heart disease included angina pectoris, myocardial infarction, and ischemic heart disease. Stroke encompassed subarachnoid hemorrhage, intracerebral hemorrhage, other non-traumatic intracranial hemorrhage, cerebral infarction, and stroke. Valvular heart disease included non-rheumatic mitral valve disorders, non-rheumatic aortic valve disorders, non-rheumatic tricuspid valve disorders, multiple valve diseases, other endocarditis, and other valve disorders. The history of these diseases was determined by the date of the first diagnosis in health-related records. The corresponding ICD-10 codes for these conditions are listed in Supplementary Table 1. All other information was collected through standardized questionnaires. Race was categorized as White, Black, Asian, or other ethnicities. Education level was classified as college or university degree, A or O levels, other qualifications, or none of the above. Employment status was categorized as employed or not employed.

### Proteomics assays

Plasma samples were obtained from more than 50,000 individuals during baseline assessment. Each sample was collected in an EDTA tube, subsequently subjected to centrifugation, and stored at -80 °C. Between 2021 and 2022, plasma samples were sent to Olink Analytical Services and analyzed using the antibody-based OlinkTM Explore 3072 proximity extension assay technology for proteomics analysis [21, 22]. In order to maintain the integrity of protein data, rigorous quality control protocols were established including careful selection of samples, importation of data, exclusion of individuals, removal of unqualified data, and exclusion of potential sample swaps. The coefficient of variation remains below 20% within groups and below 10% between groups [23]. In addition, the protein expression was presented as normalized protein expression (NPX) values defined by Olink [21, 22]. Proteins with a missing rate ≥ 20% were excluded from the study (*n* = 12). From the total study population, a subset of participants with available Olink proteomic data was included for the development of the protein risk score (*n* = 49,887). We analyzed data for 2,911 unique proteins. This proteomic sub-cohort consisted of 22,873 men and 27,014 women.

### AA protein risk score

Of the 22,873 male participants, the 19,262 from England were randomly split into a training set (*n* = 13,483) and an internal validation set (*n* = 5,779) in a 7:3 ratio, while the 3,611 from Scotland and Wales constituted the independent validation cohort. Of the 27,014 female participants, the 22,980 from England were randomly split into a training set (*n* = 16,085) and an internal validation set (*n* = 6,895) in a 7:3 ratio, while the 3,611 from Scotland and Wales constituted the independent validation cohort. Missing protein measurements were imputed with the minimum value. The details of this data imputation are presented in Supplementary Tables 3 and 4. We applied least absolute shrinkage and selection operator (LASSO) Cox regression to the training set to identify the most predictive proteins, with the penalty term for the objective function tuned via the entire training set and 10-fold cross-validation to derive the final coefficients (Supplementary Table 5). Finally, the protein risk score was calculated based on the following formula: the sum of (Coefficient [Protein 1] × Measurement [Protein 1]) + (Coefficient [Protein 2] × Measurement [Protein 2]) + … + (Coefficient [Protein n] × Measurement [Protein n]). To assess the potential influence of the missing value imputation method on LASSO protein selection results, we performed a sensitivity analysis. Specifically, missing protein measurements were imputed using K-Nearest Neighbors (KNN) imputation, and LASSO Cox regression was re-run on the same training set to compare the consistency of the proteins selected and their corresponding coefficients between the two imputation methods. The results of the sensitivity analysis are presented in Supplementary Table 6.

### Statistical analysis

Baseline features were reported as medians with interquartile ranges (IQR) for continuous variables and as frequencies (%) for categorical ones. Incidence rates of AA were presented per 1,000 person-years separately for male and female participants. Additionally, Kaplan–Meier curves were plotted to depict the cumulative incidence of AA events, using age as the time scale. We employed the Kaplan-Meier method, stratified by sex, to estimate the cumulative incidence of AA. Log-rank tests were then used to compare the cumulative incidence between participants with and without each of the eight clinical risk factors. We used Cox proportional hazards models to estimate hazard ratios (HRs) and 95% confidence intervals (CIs) for the associations between clinical risk factors and incident AA, separately in male and female participant cohorts. Model 1 was adjusted for age, SES, smoking status, obesity, hypertension, hyperlipidemia, coronary heart disease, stroke, and valvular heart disease. Model 2 was further fully adjusted for the covariates in Model 1 plus the use of antihypertensive and lipid-lowering medications.

We further estimated the sex-specific 5-, 10-, and 15-year population attributable (PAR) risks for clinical risk factors significantly associated with AA in the survival analysis. We used multivariable Cox proportional hazard model to examine the associations of proteins with incident AA. Hazard ratios (HR) and 95% confidence intervals (CI) were calculated. All models were adjusted for age. The Bonferroni correction method was used for multiple testing. Significant associations were determined when two-tailed p-values after Bonferroni correction were less than 0.05. Proteins that were significantly associated with AA risk in the prospective multivariable Cox proportional hazards models were included for analysis in g: Profiler. To control for multiple testing, the Benjamini-Hochberg method was applied, and results with a false discovery rate (FDR) below 0.05 were considered statistically significant [24, 25]. The enrichment analysis was performed based on Reactome.

Principal component analysis (PCA) was applied to reduce the dimensionality of all protein constituents within each pathway. A weighted score for each pathway was then generated from the first principal component, which captured the maximum variance and was used to represent that specific pathway. Subsequently, this weighted score was used in mediation analyses to investigate how the Reactome pathways significantly associated with AA mediate the relationship between the clinical risk factor with the strongest attributable risk and incident AA.

In the internal and independent validation cohort, we employed Cox proportional hazards models to assess the association between the protein risk score and the risk of incident AA within each sex-specific cohort. First, we calculated the hazard ratio (HR) and 95% confidence interval (CI) for incident AA per 1-standard deviation (SD) increase in the protein risk score. Furthermore, we evaluated the predictive performance of three nested models: a base model adjusted for age only, a model further incorporating clinical risk factors, and a final model that additionally included the protein risk score. The discriminatory ability of these models was assessed using Harrell’s C-statistic.

To ensure the robustness of the findings, we performed several sensitivity analyses. First, to assess the potential influence of competing risks on the study results, we conducted sensitivity analyses using the Fine-Gray competing risk model, with all-cause mortality specified as the competing event, separately for the associations between traditional clinical risk factors and incident AA risk and between sex-specific protein risk scores and incident AA risk, with covariate adjustment consistent with Model 2. Second, to further investigate the potential heterogeneity in associations between clinical risk factors and AA subtypes, we performed a sensitivity analysis evaluating the associations between the aforementioned clinical risk factors and the risk of AAA or TAA separately in male and female participants, with covariate adjustment consistent with the primary analysis.

All statistical analyses were performed using R software.

## Results

### Participant characteristics

The study initially included a total of 471,660 participants free of AA or AD at baseline, comprising 214,149 men and 257,511 women. Compared to participants who did not develop AA during follow-up, those with incident AA were older, had lower SES, and a higher prevalence of obesity and smoking. Additionally, individuals who developed AA had a higher prevalence of hypertension, hyperlipidemia, coronary heart disease, stroke, valvular heart disease, and use of lipid-lowering and antihypertensive medications. These trends were consistent in both men and women (Table 1).

**Table 1:** Baseline characteristics of study participants by incident AA status in men and women

	Male			Female		
	Non-AA (*n* = 214,039)	AA (*n* = 2955)	*P*-values	Non-AA (*n* = 256,571)	AA (*n* = 788)	*P*-values
**Age**,** y**	56.7 ± 8.2	62.4 ± 5.8	< 0.001	56.3 ± 8.0	62.1 ± 6.0	< 0.001
**Low SES**	106,860 (50.6)	1586 (53.8)	< 0.001	127,511 (49.7)	434 (55.3)	< 0.001
**Education**			< 0.001			< 0.001
College or University degree	72,496 (34.2)	660 (22.4)		80,969 (31.5)	171 (22.0)	
A, O levels and CSEs or equivalent	73,501 (34.8)	927 (31.5)		105,567 (41.1)	270 (34.4)	
NVQ\HND\HNC\Other professional qualifications	28,953 (13.7)	505 (17.1)		26,577 (10.3)	96 (12.3)	
None of the above	36,253 (17.1)	854 (29.1)		43,624 (17.0)	247 (31.3)	
**Unemployment**	14,549 (6.9)	247 (8.4)	< 0.001	22,265 (8.7)	58 (7.4)	< 0.001
**Race**			< 0.001			< 0.001
White	200,500 (94.9)	2883 (97.9)		243,724 (94.9)	763 (97.3)	
Asian	5015 (2.4)	26 (0.9)		4865 (1.9)	6 (0.8)	
Black or Black British	2986 (1.4)	20 (0.7)		4129 (1.6)	9 (1.1)	
Other ethnic	2702 (1.3)	17 (0.5)		4009 (1.6)	6 (0.8)	
**Obesity**	53,411 (25.3)	973 (33.0)	< 0.001	60,359 (23.5)	217 (27.8)	< 0.001
**Current smoke**	25,722 (12.2)	717 (24.2)	< 0.001	22,546 (8.8)	208 (26.0)	< 0.001
**Hypertension**	66,953 (30.8)	1534 (50.9)	< 0.001	62,360 (24.3)	368 (46.9)	< 0.001
**Hyperlipidemia**	39,763 (18.8)	1021 (34.6)	< 0.001	29,945 (11.7)	204 (26.0)	< 0.001
**Coronary heart disease**	16,426 (7.8)	624 (21.2)	< 0.001	7832 (3.1)	101 (12.8)	< 0.001
**Stroke**	4204 (2.0)	140 (4.8)	< 0.001	3087 (1.2)	35 (4.6)	< 0.001
**Valvular heart disease**	1596 (0.8)	85 (3.1)	< 0.001	1370 (0.5)	33 (4.2)	< 0.001
**Blood pressure medication**	51,105 (24.2)	1326 (45.0)	< 0.001	44,897 (17.5)	308 (39.1)	< 0.001
**Cholesterol lowering medication**	49,404 (23.4)	1295 (44.0)	< 0.001	33,394 (13.0)	230 (29.2)	< 0.001

SES: socioeconomic status; Low SES: High Townsend deprivation index (TDI); Obesity: BMI >=30.0kg/m2; AA: Aortic aneurysm

### Incidence of aortic aneurysm in men and women

**Fig. 1:**
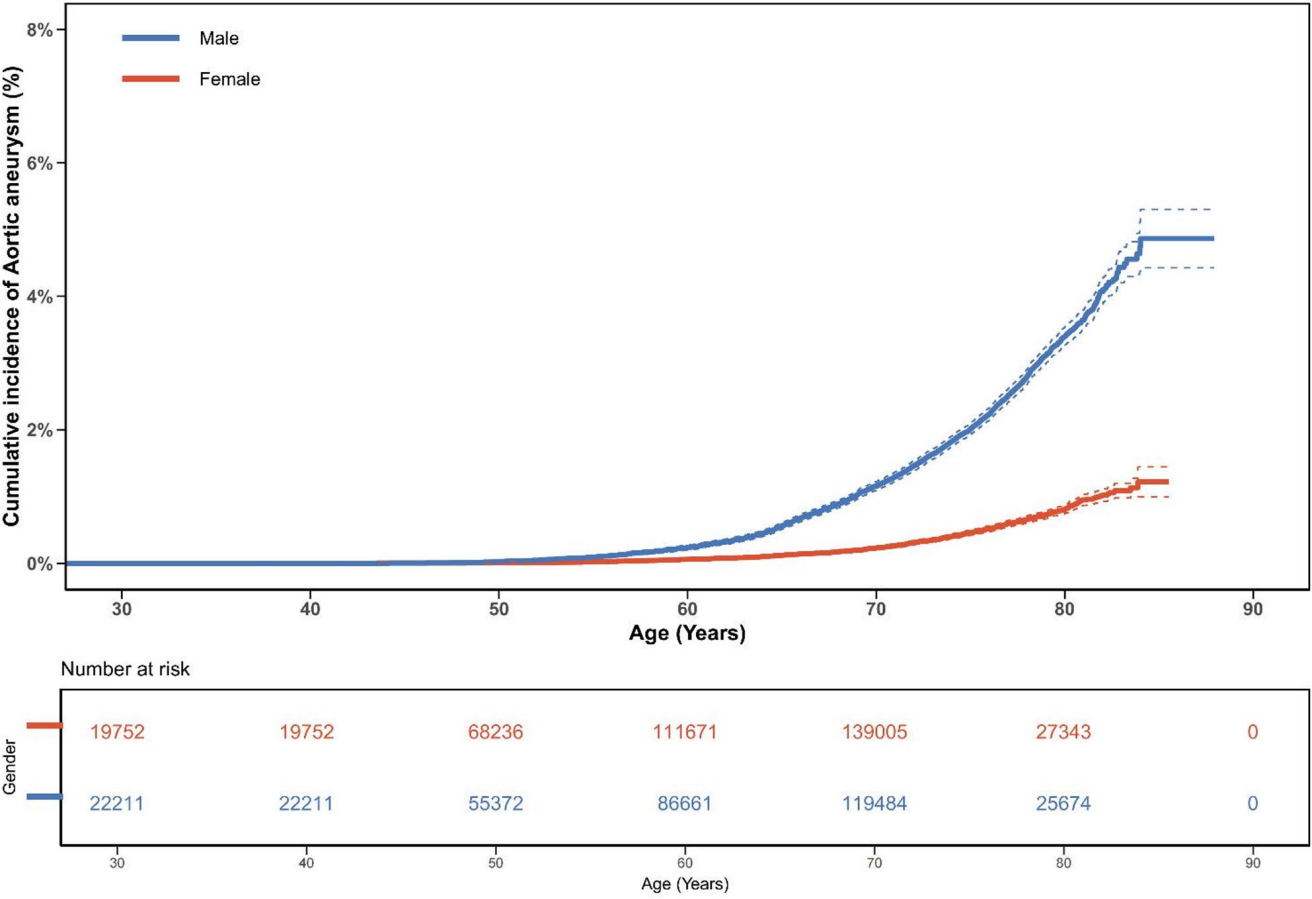
Cumulative incidence of AA in men and women according to age. Age is represented on the X-axis, and cumulative incidence is shown on the Y-axis. AA: Aortic aneurysm

Over a median follow-up of 13.74 years, 3,730 incident AA events were recorded (2,946 in men and 784 in women). The incidence rate was substantially higher in men (1.0 per 1,000 person-years) than in women (0.2 per 1,000 person-years). As shown in Fig. 1, the cumulative incidence of AA increased with age in both sexes, with a notably steeper acceleration observed in men after the age of 55.

### Association between risk factors and incident AA

**Fig. 2:**
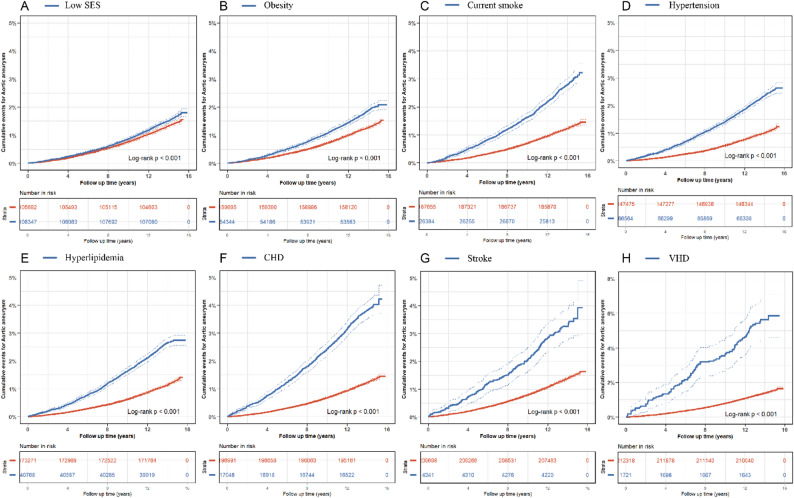
Cumulative incidence of AA by clinical risk in men. SES (**A**); Obesity (**B**); Smoke (**C**); Hypertension (**D**); Hyperlipidemia (**E**); CHD (**F**); Stroke (**G**); VHD (**H**). SES: socioeconomic status; CHD: Coronary heart disease; VHD: Valvular heart disease

**Fig. 3:**
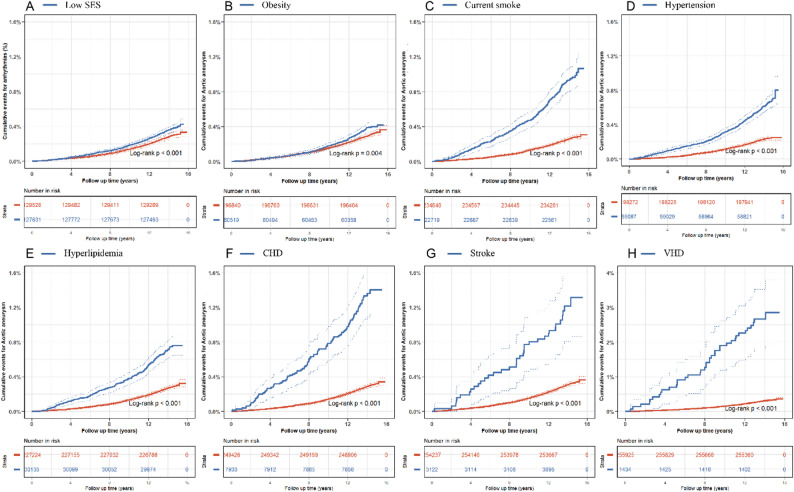
Cumulative incidence of AA by clinical risk in women. SES (**A**); Obesity (**B**); Smoke (**C**); Hypertension (**D**); Hyperlipidemia (**E**); CHD (**F**); Stroke (**G**); VHD (**H**). SES: socioeconomic status; CHD: Coronary heart disease; VHD: Valvular heart disease

**Fig. 4:**
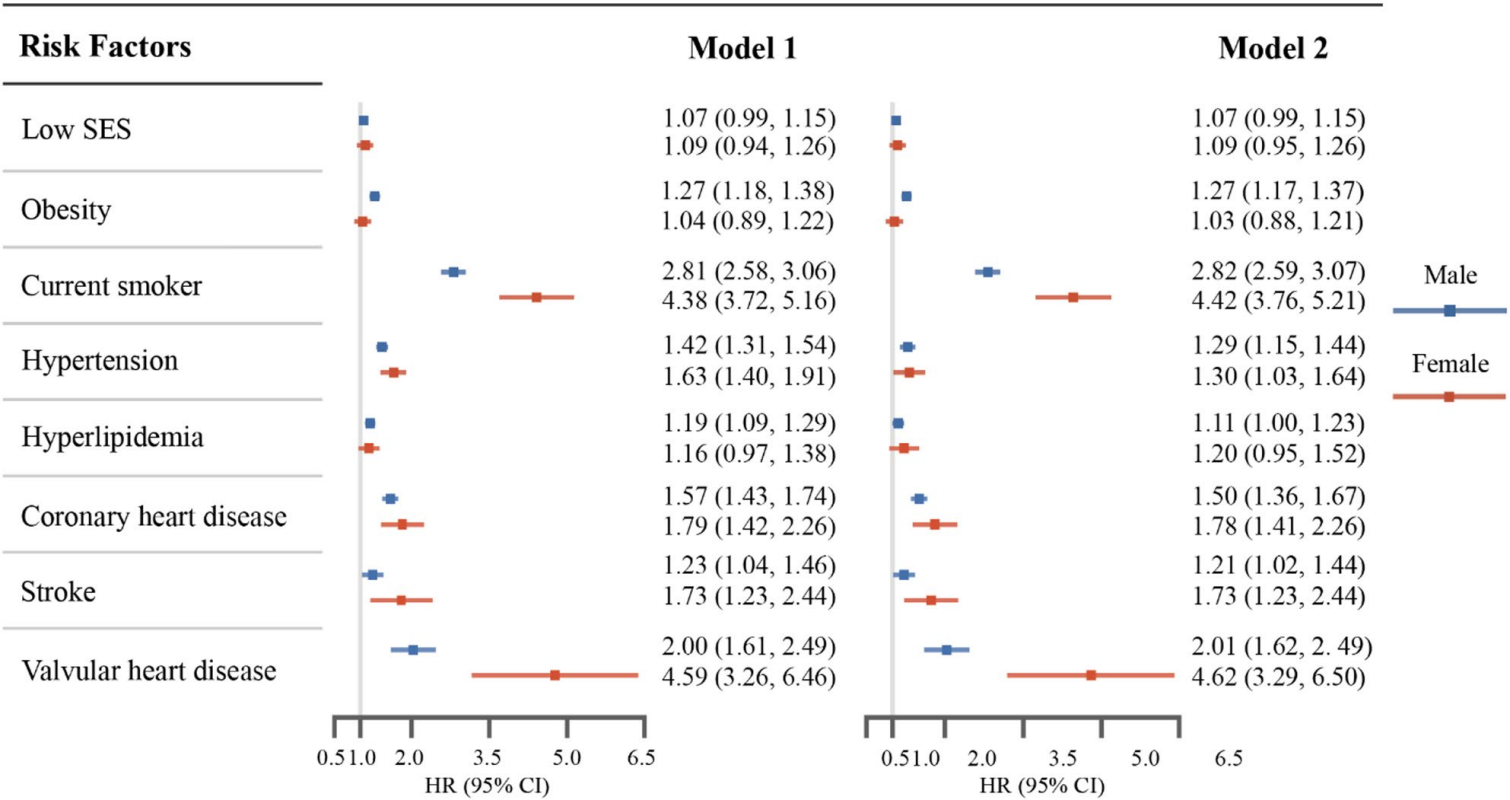
Association between clinical risk factors and incident AA. Model 1 was adjusted for age, SES, smoking status, obesity, hypertension, hyperlipidemia, coronary heart disease, stroke, and valvular heart disease. Model 2 was further adjusted for the use of antihypertensive and lipid-lowering medications. HR, hazard ratio; CI, confidence interval

The sex-specific cumulative incidence of AA is shown in Figs. 2 and 3. An initial univariable screening via Kaplan-Meier analysis and log-rank tests revealed that all eight risk factors were universally and significantly associated with an increased AA risk (*p* < 0.05) in both males and females. Figure 4 presents the results of the fully adjusted Cox model. With the exception of SES, all traditional clinical risk factors were significantly associated with an elevated risk of incident AA in men. In contrast to the findings in men, neither obesity nor hyperlipidemia showed a significant association with AA risk in women. No significant association was observed between SES and the risk of incident AA in women, a finding consistent with that in men. Notably, smoking and VHD were the two strongest risk factors for AA in both men and women, although the magnitude of their impact differed. The relative risks associated with smoking (Female: HR 4.42, 95% CI 3.76–5.21; Male: HR 2.82, 95% CI 2.59–3.07) and VHD (Female: HR 4.62, 95% CI 3.29–6.50; Male: HR 2.01, 95% CI 1.62–2.49) were significantly higher in women than in men. Furthermore, the magnitude of risk associated with other shared factors was also consistently greater in women than in men.

The sensitivity analysis results demonstrated that the findings from the competing risk model, with all-cause mortality as the competing event, were highly consistent with those obtained from the Cox proportional hazards model, indicating that the primary conclusions are robust (Supplementary Table 7). Sensitivity analyses examining AA subtypes revealed heterogeneity in the associations between risk factors and TAA versus AAA (Supplementary Tables 8 and 9). In both sexes, the association between smoking and AAA risk was stronger than that observed for composite AA, whereas the association with TAA was non-significant in men but remained significant in women. Valvular heart disease showed stronger associations with TAA risk in both sexes, with no significant association observed for AAA. Obesity, hyperlipidemia, coronary heart disease, and stroke showed stronger associations with AAA than TAA in men. In women, coronary heart disease was primarily associated with AAA risk, whereas stroke showed significant associations with both AAA and TAA. These findings suggest subtype-specific heterogeneity in the effects of different risk factors on AAA and TAA, further highlighting the pathophysiological distinctions between AAA and TAA.

### PAR for incident AA in men and women

**Fig. 5:**
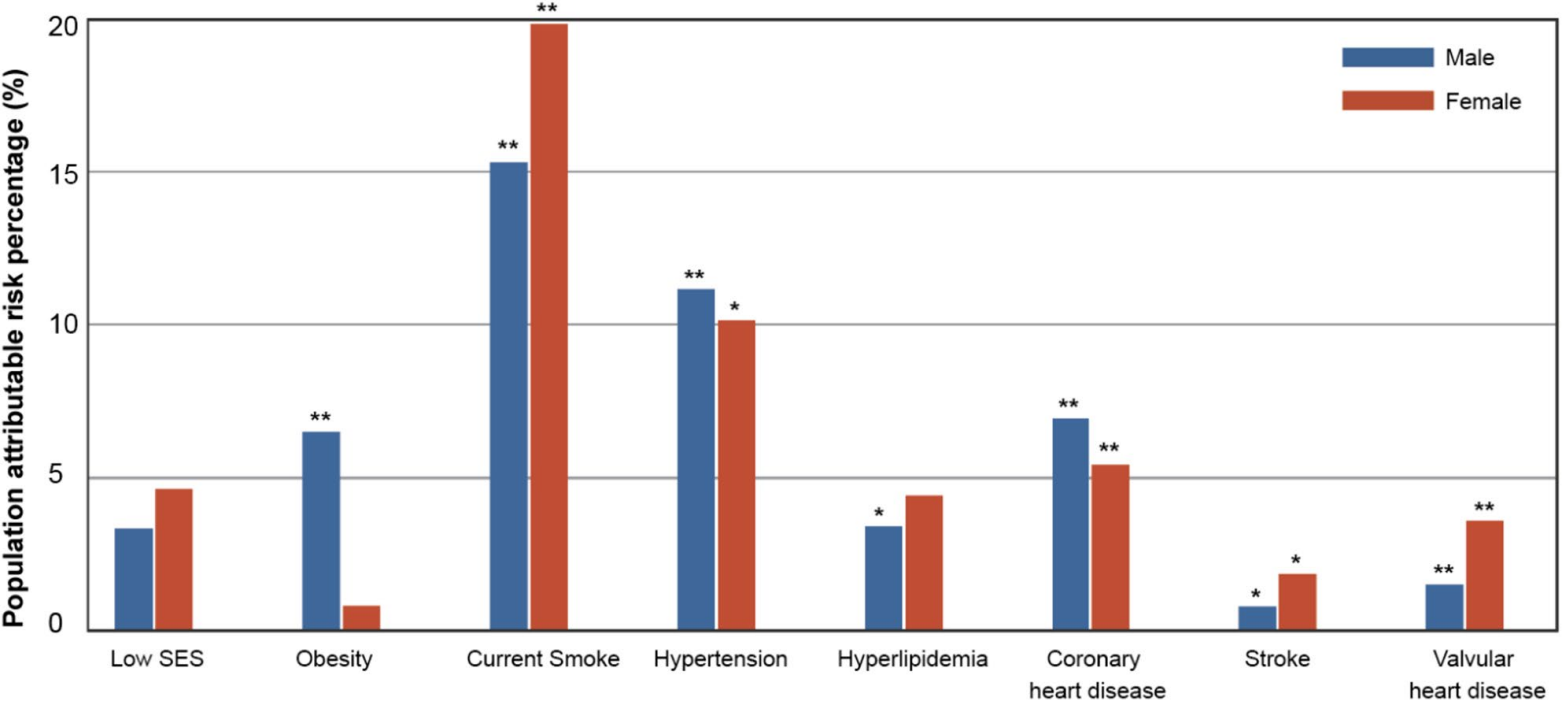
15-year PAR of Risk Factors in Men and Women. Model was adjusted for age, SES, smoking status, obesity, hypertension, hyperlipidemia, coronary heart disease, stroke, valvular heart disease, antihypertensive and lipid-lowering medications. PAR: population attributable risk; SES: socioeconomic status. *: *p* < 0.05; **: *p* < 0.001

Supplementary Tables 10 and 11 present the population attributable risk (PAR) proportions for individual risk factors at 5, 10, and 15 years of follow-up in men and women, respectively. The PAR estimates remained relatively stable across follow-up periods. As shown in Fig. 5, smoking was the strongest attributable factor in both sexes, accounting for 15.3% of AA risk in men and 19.8% in women, followed by hypertension (11.2% in men and 10.2% in women). Additionally, obesity and hyperlipidemia combined accounted for 10.0% of AA risk in men, whereas the PAR for these two factors was not statistically significant in women. The PAR for socioeconomic status was non-significant in both sexes, consistent with the Cox regression findings (Fig. 4). Collectively, the identified risk factors accounted for 45.9% and 45.3% of the population-level risk for incident AA in men and women, respectively.

### Association between protein levels and incident AA in men and women

**Fig. 6:**
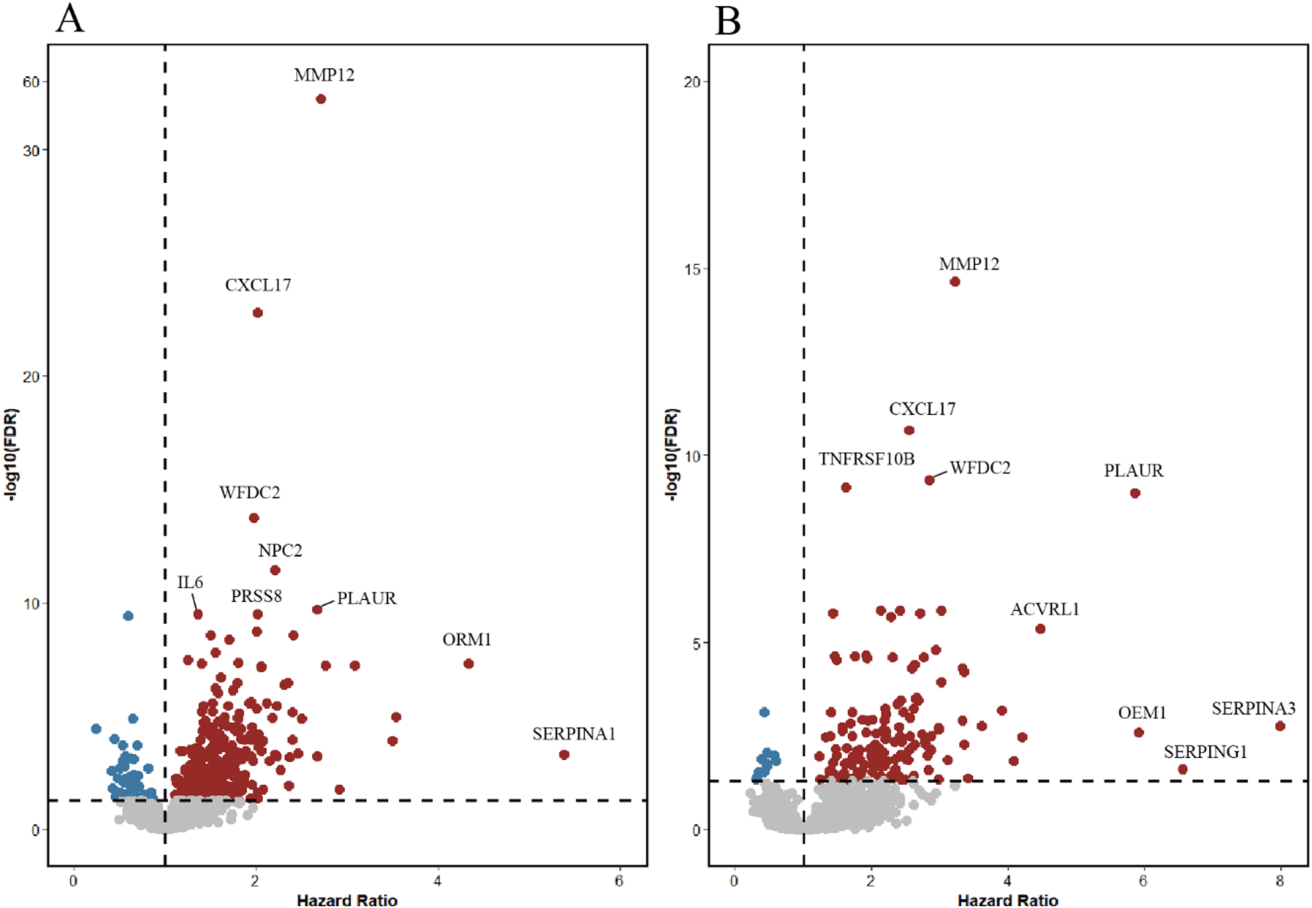
Association between protein expression levels and hazard ratios for AA in men (**A**) and women (**B**). Model was adjusted for age

Multivariable Cox proportional hazards regression models were employed to examine the associations between plasma protein levels and incident AA risk among participants without a diagnosis of AA or AD at baseline. In age-adjusted models, 395 and 166 plasma proteins were significantly associated (Bonferroni-adjusted *P* < 0.05) with incident AA risk in men and women, respectively (Fig. 6; Supplementary Tables 12 and 13). In men, 336 proteins were positively associated with AA risk and 59 showed inverse associations. In women, 154 proteins were positively associated with AA risk and 12 showed inverse associations. A core set of 126 proteins was significantly associated with AA risk in both sexes, among which MMP-12, CXCL17, WFDC2, and CST3 were consistently identified as robust risk markers. Notably, among shared risk-associated proteins, a one-unit increase in plasma concentration was generally associated with a greater relative risk increase in women than in men.

### Biological function of AA-associated proteins

**Fig. 7:**
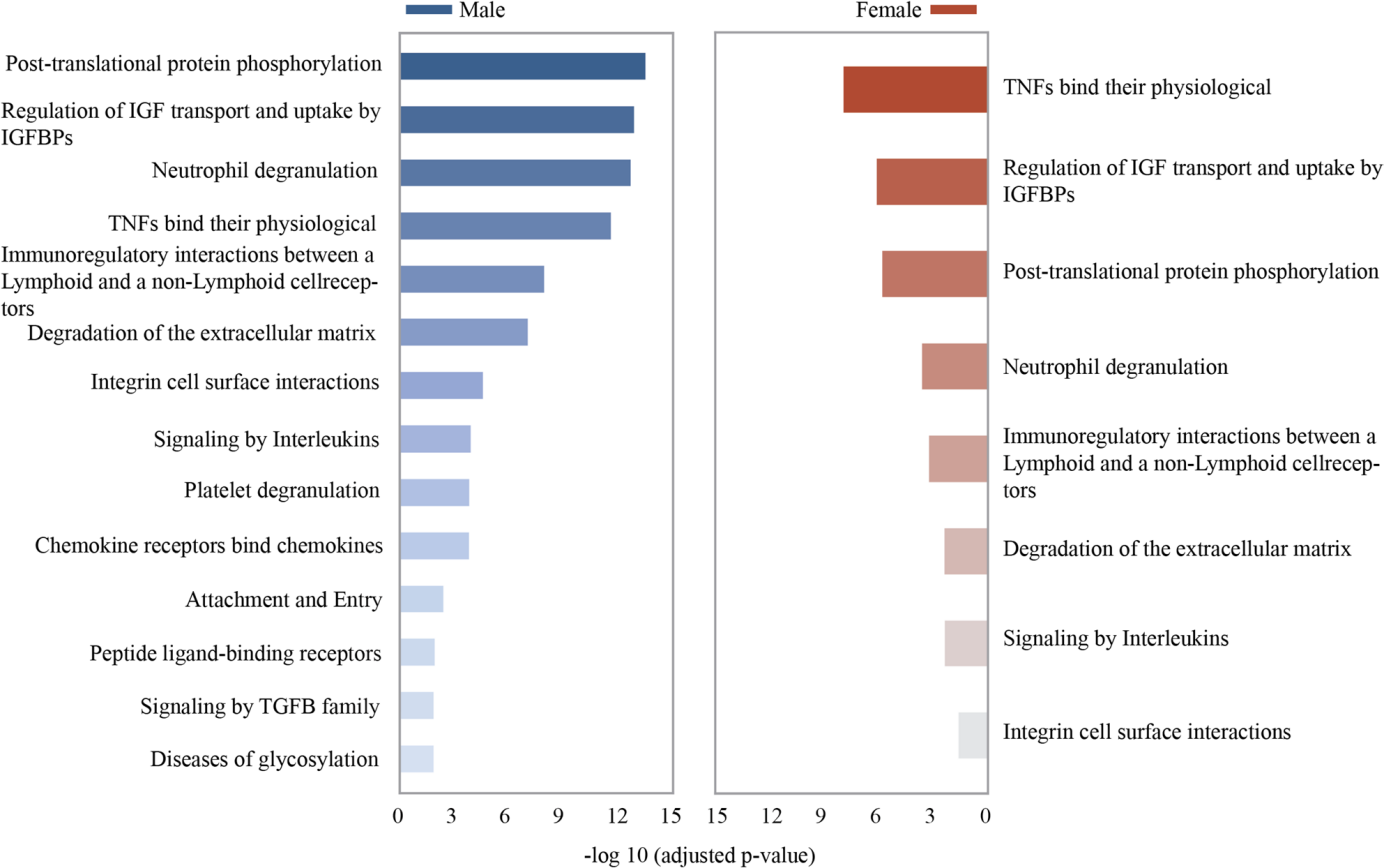
Enrichment analysis results for Reactome pathways in men and women. IGF: insulin-like growth factor; IGFBP: insulin-like growth factor binding protein; TNF: tumor necrosis factor; TGFB: Transforming Growth Factor-Beta

Prospective analyses identified 395 and 166 proteins significantly associated with incident AA risk in men and women, respectively. We incorporated these proteins into g: Profiler for Reactome pathway clustering, separately for each sex. The pathway enrichment results are presented in Fig. 7 and Supplementary Tables 14 and 15. The results showed that female-specific AA-associated pathways were primarily enriched in immune and inflammatory responses (e.g., “TNFs bind their physiological receptors”), pathways related to the regulation of cellular growth factors (e.g., “Regulation of IGF transport and uptake by IGFBPs”), and extracellular matrix organization (e.g., “Degradation of the extracellular matrix”). In males, the vast majority of pathways were consistent with those associated with AA in females, with some additional pathways related to coagulation and vascular processes, as well as glycosylation diseases. Furthermore, all AA-associated pathways enriched in females were contained within the set of pathways identified in males. Overall, the immune system and the regulation of cellular growth factors were the most relevant pathways for AA, a finding observed consistently in both men and women.

### Mediating pathways between smoking or hypertension and incident AA

Mediation analyses were conducted separately in men and women to evaluate the proportion of the smoking- and hypertension-AA associations potentially explained by AA-related Reactome pathways (Tables 2 and 3). For smoking, the “Degradation of the extracellular matrix” pathway emerged as the dominant potential mediator in both sexes, explaining 37.1% of the risk association in men and 26.1% in women, suggesting a shared structural degradation mechanism that may underlie smoking-related AA risk.

In contrast, the mediation profile for hypertension suggested marked sex divergence. In men, ECM degradation remained the primary candidate mediating pathway, potentially accounting for 49.0% of the hypertension-AA association. In women, however, hypertension risk may be predominantly explained by metabolic and growth factor regulation pathways rather than structural degradation. Specifically, “Regulation of IGF transport and uptake by IGFBPs” explained the largest proportion (47.5%), followed by “Post-translational protein phosphorylation” (46.7%) and “Signaling by Interleukins” (44.3%). It should be noted that, owing to overlapping proteins enriched across pathways, the sum of individual mediation proportions does not equal 100%.

**Table 2:** Mediation Analysis (Reactome) of the pathway between Smoking or hypertension and AA in men

Pathway	Smoke	Hypertension
Post-translational protein phosphorylation	27.6% (20.4%-40.1%)	13.9% (9.6%-21.4%)
Regulation of IGF transport and uptake by IGFBPs	25.7% (19.5%-37.7%)	34.5% (25.3%-48.5%)
Neutrophil degranulation	26.5% (19.2%-39.5%)	22.4% (15.8%-31.2%)
TNFs bind their physiological receptors	17.1% (12.6%-25.3%)	27.2% (19.9%-39.3%)
Immunoregulatory interactions between a Lymphoid and a non-Lymphoid cell	16.1% (11.5%-23.9%)	24.3% (17.7%-34.3%)
Degradation of the extracellular matrix	37.1% (27.0%-54.4%)	49.0% (36.9%-71.9%)
Integrin cell surface interactions	13.9% (9.6%-21.4%)	20.4% (14.6%-29.2%)
Signaling by Interleukins	25.9% (18.7%-39.5%)	23.9% (16.7%-33.4%)
Platelet degranulation	17.2% (11.9%-26.8%)	19.5% (13.5%-28.5)
Chemokine receptors bind chemokines	10.9% (7.6%-16.8%)	22.3% (15.7%-31.0)
Attachment and Entry	12.2% (8.5%-18.2%)	29.3% (20.7%-43.8%)
Peptide ligand-binding receptors	13.2% (9.4%-19.6%)	28.1% (20.2%-40.4%)
Signaling by TGFB family members	8.7% (5.7%-13.4%)	23.6% (16.3%-33.7%)
Diseases of glycosylation	7.7% (4.9%-12.0%)	20.7% (13.2%-28.2%)

IGF: insulin-like growth factor; IGFBP: insulin-like growth factor binding protein; TNF: tumor necrosis factor; TGFB: Transforming Growth Factor-Beta

**Table 3:** Mediation Analysis (Reactome) of the pathway between Smoking or hypertension and AA in women

Pathway	Smoke	Hypertension
TNFs bind their physiological receptors	9.2% (6.4%-16.4%)	33.9% (20.4%-68.7%)
Regulation of IGF transport and uptake by IGFBPs	22.0% (15.7%-32.7%)	47.5% (28.0%-80.2%)
Post-translational protein phosphorylation	20.0% (15.0%-32.2%)	46.7% (28.4%-95.3%)
Neutrophil degranulation	23.5% (16.5%-34.6%)	31.6% (18.6%-70.1%)
Immunoregulatory interactions between a Lymphoid and a non-Lymphoid cell	11.2% (7.6%-17.1%)	35.6% (21.7%-82.1%)
Degradation of the extracellular matrix	26.1% (18.8%-44.0%)	33.9% (20.4%-68.7%)
Signaling by Interleukins	13.9% (9.7%-21.0%)	44.3% (27.5%-87.6%)
Integrin cell surface interactions	12.4% (8.4%-18.1%)	34.1% (20.5%-67.2%)

IGF: insulin-like growth factor; IGFBP: insulin-like growth factor binding protein; TNF: tumor necrosis factor

### Association between AA protein risk score and the risk of incident AA

Proteins demonstrated to be significantly associated with AA risk through Cox proportional hazards models were incorporated into the construction of a protein risk score. In the training cohort (Male: *n* = 13,483; Female: *n* = 16,085), the LASSO method identified 14 and 13 proteins from 395 to 166 circulating plasma proteins, respectively, which were used to construct male-specific and female-specific AA protein risk scores (Table S4). Among these, four proteins—MMP12, CXCL17, IL6, and NT-proBNP—were significantly associated with AA in both sexes. Ten proteins were significantly associated with AA risk exclusively in males, and nine proteins were significantly associated with AA exclusively in females. These results indicate that the proteins constituting the AA protein risk profile differ significantly between males and females.

Table 4 shows the association between the protein risk score and incident AA risk in the internal and independent validation cohort. After adjusting for clinical risk factors and age, the AA protein risk score showed significant positive associations with AA risk in both the internal validation set (per SD increment: Male: HR, 1.86, 95% CI 1.50–2.32; Female: HR, 1.50, 95% CI 1.06–2.12) and the independent validation cohort (per SD increment: Male: HR, 2.02, 95% CI 1.53–2.68; Female: HR, 1.89, 95% CI 1.19-3.00). The sensitivity analysis results demonstrated that the findings from the Fine-Gray competing risk model were highly consistent with those obtained from the Cox proportional hazards model (Supplementary Table 16).

**Table 4:** Association between Protein risk score and risk of AA

	Adjust variables	HR per SD protein risk score in men (95% CI)	HR per SD protein risk score in women (95% CI)
Internal validation set	Age	2.04 (1.70, 2.45)	1.95 (1.51, 2.51)
	Age and Clinical risks factors	1.86 (1.50, 2.32)	1.50 (1.06, 2.12)
Independent validation cohort	Age	2.32 (1.85, 2.90)	2.05 (1.35, 3.10)
	Age and Clinical risks factors	2.02 (1.53, 2.68)	1.89 (1.19, 3.00)

HR, hazard ratio; CI, confidence interval; Clinical risks factors including SES, smoking status, obesity, hypertension, hyperlipidemia, coronary heart disease, stroke and valvular heart disease

### Discrimination and reclassification ability assessment for the AA protein risk score

Table 5 presents the discriminative ability of the predictive models in both the internal and independent validation cohorts. Specifically, in the internal validation set, the baseline age-only model yielded a C-statistic of 0.724 for men and 0.766 for women. The inclusion of clinical risk factors improved these values to 0.784 and 0.816, respectively. The final model, integrating age, clinical factors, and the protein risk score, achieved the highest predictive performance, with C-statistics reaching 0.802 in men and 0.832 in women.

In the independent validation cohort, the protein risk score demonstrated robust incremental value. Compared to the clinical risk model (C-statistic: Men 0.784; Women 0.790), the addition of the protein risk score further enhanced discrimination, yielding a final C-statistic of 0.809 (95% CI 0.745–0.872) for men and 0.832 (95% CI 0.775–0.839) for women.

In summary, the integration of the sex-specific protein risk score with traditional clinical factors consistently maximized predictive accuracy for incident AA across both cohorts and sexes. In the independent validation cohort, model calibration performance was assessed using calibration slopes (Supplementary Table 17). The addition of the protein risk score improved calibration slopes in both men and women, suggesting that integrating the protein risk score enhances model calibration performance. The relatively lower calibration slope observed in women primarily reflects the objective limitation of the limited number of AA events in the female cohort. A sensitivity analysis using KNN imputation yielded a protein risk score with broadly consistent improvement in predictive performance over the clinical risk factor model in both the internal and independent external validation cohorts, compared with the primary analysis (Supplementary Table 18).

**Table 5:** Performance of Risk Prediction Models for Incident AA in the Internal Validation Set and the Independent validation cohort

Model	C statistic (men)	C statistic (women)
**Internal validation set**		
Age	0.724 (0.674–0.775)	0.766 (0.707–0.825)
Age + protein risk score	0.783 (0.728–0.837)	0.806 (0.741–0.870)
Age + Clinical risk factors	0.784 (0.736–0.844)	0.816 (0.755–0.878)
Age + Clinical risk factors + protein risk score	0.802 (0.753–0.805)	0.832 (0.775–0.889)
**Independent validation cohort**		
Age	0.702 (0.636–0.769)	0.684 (0.560–0.800)
Age + protein risk score	0.793 (0.728–0.858)	0.774 (0.665–0.883)
Age + Clinical risk factors	0.784 (0.719–0.848)	0.790 (0.780–0.830)
Age + Clinical risk factors + protein risk score	0.809 (0.745–0.872)	0.832 (0.775–0.839)

Clinical risks factors including SES, smoking status, obesity, hypertension, hyperlipidemia, coronary heart disease, stroke and valvular heart disease

## Discussion

In this large-scale prospective cohort study, we systematically characterized the clinical and proteomic risk landscapes of AA and examined their sex-specific differences. Although the incidence of AA was lower in women than in men, women faced a comparatively higher relative risk upon exposure to the same risk factors. Among the clinical risk factors examined, smoking and hypertension contributed most substantially to AA susceptibility in both sexes. Mediation analysis suggested that the associations between smoking or hypertension and AA may be partially explained by pathways such as “Degradation of the extracellular matrix” and “Regulation of Insulin-like Growth Factor (IGF) transport and uptake by Insulin-like Growth Factor Binding Proteins (IGFBPs)”. Furthermore, sex-specific protein risk scores were developed from multiple plasma proteins. The proteins contributing to AA risk showed a high degree of sex specificity: only 4 proteins were shared between sexes, while 10 were exclusive to men and 9 were exclusive to women. Ultimately, a predictive model integrating traditional clinical risk factors with the sex-specific protein risk scores demonstrated superior discriminatory performance in the independent validation cohorts (C-statistic: men: 0.809; women: 0.832).

Prior studies have examined the associations between traditional clinical risk factors and AA risk [14–31]. However, most were conducted in mixed-sex populations without a sex-stratified analytical framework. The present study extends this evidence by systematically characterizing the sex-specific associations between traditional risk factors and AA. The absolute incidence of AA in men was approximately five times that in women. In men, obesity, smoking, hypertension, hyperlipidemia, coronary heart disease (CHD), stroke, and valvular heart disease (VHD) were each associated with an elevated AA risk. In women, all of these factors—with the exception of obesity and hyperlipidemia—were similarly associated with increased risk. Notably, smoking and VHD were the two strongest shared risk factors across sexes, yet both conferred a substantially higher relative risk in women than in men. Current smoking was associated with a 2.82-fold increase in AA risk in men and a 4.42-fold increase in women, relative to never-smokers. VHD was the strongest individual risk factor in women (HR: 4.62, 95% CI: 3.29–6.50), with a significantly greater magnitude of association than in men (HR: 2.01, 95% CI: 1.62–2.49). These findings suggest that aortic surveillance may be clinically warranted in patients with valvular disease, particularly in women.

The stronger associations of CHD and stroke with AA risk in women suggest that female AA may be accompanied by more widespread systemic vascular pathology. In contrast, obesity and dyslipidemia appeared to operate as male-specific risk factors. Collectively, these findings indicate that the pathophysiological mechanisms underlying AA may differ substantially between sexes, underscoring the importance of elucidating the relevant molecular mechanisms through proteomic pathway analysis.

To quantify disease burden, population attributable risk (PAR) was estimated separately for men and women. Smoking and hypertension were identified as the primary attributable risk factors, with smoking being the strongest contributor (15.3% in men and 19.8% in women), followed by hypertension (11.2% in men and 10.2% in women). Mediation analysis was further employed to characterize the proteomic pathways potentially underlying the effects of smoking and hypertension on AA risk, with particular attention to sex differences. The results indicated that “Degradation of the extracellular matrix,” as a shared structural mechanism, may account for the highest proportion of smoking-related AA risk in both men (37.1%) and women (26.1%). This suggests that regardless of sex, smoking may primarily influence AA risk through proteolytic destruction of the aortic wall. However, a distinct sexual dimorphism emerged in the mechanism of hypertension. In men, the association between hypertension and AA may be partly explained by structural ECM degradation (mediation proportion up to 49.0%). In contrast, the association between hypertension and AA in women may be primarily explained by metabolic and growth factor regulation pathways. Specifically, the “Regulation of Insulin-like Growth Factor (IGF) transport and uptake by Insulin-like Growth Factor Binding Proteins (IGFBPs)” pathway may explain the largest proportion of hypertension-associated AA risk in women (47.5%). These findings suggest that while smoking may operate through a shared matrix degradation pathway in both sexes, the vascular response to hemodynamic stress appears to exhibit substantial sex divergence. Moreover, as traditional clinical risk factors collectively accounted for only 45.9% and 45.3% of the population-level risk for incident AA in men and women, respectively, these factors alone are insufficient to fully capture the residual risk of AA, highlighting the need for novel proteomic markers to improve risk stratification.

We constructed protein risk scores to adequately capture the residual risk of AA and identify high-risk populations. A recent study using the same dataset identified protein biomarkers for a composite outcome of AA or aortic dissection (AD), confirming that including these proteins improved prediction [17]. That study integrated protein biomarkers with demographic factors, achieving an AUC of 0.777. While valuable, that approach was limited by phenotypic heterogeneity as it did not distinguish between AA and AD. In our study, we adopted a sex-stratified strategy, given the sex-specific nature of AA risk profiles. Consequently, our model integrating the sex-specific protein score and traditional risk factors achieved C-indices of 0.809 and 0.832 in independent validation cohorts for men and women, respectively, outperforming models developed in mixed populations. This improvement in predictive performance demonstrates the distinct advantage of a sex-stratified strategy. Nevertheless, the clinical implications of the sex-specific protein risk score warrant further investigation. Currently, AA screening relies primarily on imaging-based strategies such as ultrasound. Imaging detects existing AA lesions, whereas the protein risk prediction model developed in this study aims to estimate the associated risk of future AA development; the two approaches are therefore complementary rather than mutually exclusive. We believe the potential clinical value of this model is primarily reflected in the following scenarios. First, for women and individuals without traditional risk factors, the protein risk score may serve as a complementary tool to identify high-risk individuals, thereby prioritizing targeted imaging examinations and improving screening efficiency. Second, in resource-limited healthcare settings, the protein risk score may be used to stratify individuals prior to imaging examinations, enabling more rational allocation of limited imaging resources. Third, for individuals with multiple established traditional risk factors, the protein risk score may further quantify residual risk beyond conventional risk assessment, providing additional information to support clinical decision-making. We emphasize that this model is intended to complement rather than replace existing imaging-based screening strategies, and its actual clinical value remains to be further validated through prospective interventional studies.

Our results indicate that the proteins incorporated into the risk scores exhibit significant sex differences. Among them, 10 were male-specific, 9 were female-specific, and only 4 (MMP12, CXCL17, IL-6, and NT-proBNP) were shared between sexes. This further validates the necessity of capturing AA residual risk through sex stratification. It is noteworthy that MMP12 and CXCL17 have been confirmed to have significant predictive value for AA or AD in previous studies [17]. MMP12, a macrophage elastase involved in embryonic development and tissue remodeling, is critical for elastin breakdown and matrix degradation. It is highly expressed in AAA tissues, particularly in infiltrating macrophages, and localized to regions of elastin degradation in the aortic wall [32]. CXCL17 is a mucosal chemokine that promotes inflammation and fibrosis via MRGPRX2 activation [33, 34]. A prior study showed that circulating CXCL17 levels rise with age and are associated with cardiac dysfunction; its deletion or neutralization significantly suppressed cardiac hypertrophy and fibrosis induced by aging and angiotensin II [35]. IL-6 is a pro-inflammatory cytokine that promotes endothelial dysfunction and plaque formation, leading to increased cardiovascular risk [36]. NT-proBNP is a recognized biomarker of cardiovascular injury, reflecting wall stress and dysfunction [37]. Crucially, although these proteins were associated with AA in both sexes, their contribution to the protein scores differed significantly. Among the proteins constructing the male-specific score, MMP12 dominated with the highest weight of 0.678, whereas its weight was lower in women (0.390). Additionally, among male-specific proteins, ADGRG1 contributed to the risk score comparably to the classic inflammatory factor IL-6. ADGRG1 (also known as GPR56) is an adhesion G protein-coupled receptor expressed on cytotoxic lymphocytes such as NK cells and CD8 + T cells, playing a complex regulatory role in their cytotoxic functions [38]. The prominence of MMP12 establishes matrix degradation as central to male pathology. Together, CXCL17, ADGRG1, and IL-6 reveal a pathological landscape characterized by cytotoxic immune cell infiltration and heightened inflammation. This may explain the phenomena of rapid aneurysm expansion and rupture that are more common in men [39, 40].

Distinct from males, TNFRSF10B had the highest positive coefficient in the female protein score. As a key receptor triggering the extrinsic apoptosis pathway, TNFRSF10B promotes the apoptosis of vascular smooth muscle cells (VSMCs), which are essential for maintaining the structural integrity and tone of the aortic wall. VSMC apoptosis leads to pathological remodeling and weakening of the wall, increasing the risk of aneurysmal expansion and rupture [41]. Furthermore, among female-specific proteins, NCAN showed a strong negative correlation. As a chondroitin sulfate proteoglycan, NCAN is a key structural component of the vascular extracellular matrix, contributing to hydration and viscoelasticity while facilitating the connection between smooth muscle cells and elastic fibers [42]. This inverse relationship suggests that AA risk in women may be driven by an insufficiency of protective matrix synthesis or reserve, rather than solely by matrix degradation. Finally, ALPP and PLAUR mark maladaptive remodeling processes. PLAUR is a cell surface receptor that mediates various functions of the plasminogen activation system and regulates cell adhesion and intracellular signaling through interactions with ECM components and receptors; its expression is upregulated under conditions of tissue injury or inflammation [43, 44]. Ectopic expression of ALPP is associated with cellular stress and microenvironmental calcification pressure. As an alkaline phosphatase, ALPP promotes mineralization under stress conditions by increasing inorganic phosphate and reducing pyrophosphate (an inhibitor of mineral formation), thereby fostering calcification. Moreover, previous research indicates that ALP expression in VSMCs is upregulated under endoplasmic reticulum stress via an ATF4-dependent mechanism, linking cellular stress to vascular calcification [45–47].

### Strengths and limitations

Our study has several limitations that merit consideration. First, although we employed a geographically distinct cohort (Scotland and Wales) for validation to minimize overfitting, we acknowledge that these participants were recruited under the same UK Biobank protocol as the training set. Therefore, true external validation in completely independent, multi-ethnic cohorts is warranted to further confirm the generalizability and calibration of our sex-specific protein risk scores, as current findings are primarily applicable to White European populations. Furthermore, the relatively lower calibration slope observed in the female model is primarily attributable to the limited number of AA events in women. This study is primarily intended as a proof-of-concept investigation providing preliminary evidence for sex-specific protein risk prediction, and further validation of model calibration performance in larger independent female cohorts remains warranted. Second, the UK Biobank is subject to a degree of healthy volunteer selection bias, whereby participants differ from the general population in terms of overall health status, socioeconomic characteristics, and lifestyle, which may to some extent affect the generalizability of the findings of this study. Third, given the asymptomatic latent period of early-stage AA, the incidence reported in our study may be an underestimation, as undiagnosed cases without incidental imaging findings likely remain undetected. Fourth, both the pathway enrichment analyses and mediation analyses were based on observational proteomic associations. The biological pathways identified therefore represent potentially hypothetical mechanisms rather than definitive causal evidence. Fifth, the limited number of ruptured aneurysm events precluded separate analyses of ruptured outcomes across all analyses, including both traditional risk factor analyses and proteomic analyses. Furthermore, although sensitivity analyses examining traditional clinical risk factors were conducted separately for AAA and TAA, subtype-specific proteomic analyses were not performed, as the relatively small numbers of AAA and TAA cases individually, particularly after sex stratification, would have resulted in insufficient statistical power. Therefore, our analyses focused on AA as a composite outcome. Future studies with larger sample sizes are warranted to delineate rupture-specific risk profiles and subtype-specific proteomic signatures. Sixth, the diagnosis of AA in this study was based primarily on ICD codes derived from hospital and registry data. As important clinical imaging information—such as aneurysm diameter, growth rate, and disease severity—is unavailable in the UK Biobank, and given that aneurysm size is a critical factor in the assessment of clinical risk and management decisions, this study was unable to perform further stratified analyses of risk profiles or mechanistic differences across varying degrees of AA severity, which to some extent limits the clinical interpretability of the findings. Finally, despite rigorous adjustment for a wide range of covariates, the possibility of residual confounding from unmeasured factors (such as hemodynamic parameters or genetic predisposition) cannot be entirely excluded.

## Conclusions

In conclusion, this study systematically characterized sex-specific differences in traditional clinical risk factors and proteomic risk profiles for AA. Smoking and hypertension were identified as the primary modifiable risk factors for AA, and timely intervention targeting these factors may substantially reduce AA risk, particularly in women. Additionally, mediation analysis elucidated the proteomic pathways through which smoking and hypertension may influence AA risk, revealing important sex-specific mechanistic differences. Finally, sex-specific protein risk scores were developed, and a predictive model integrating these scores with traditional clinical risk factors demonstrated strong discriminatory performance for incident AA in both men and women. Collectively, these findings provide novel insights for the identification of high-risk individuals and the advancement of precision medicine approaches to AA prevention and early detection.

## Supplementary Information

Below is the link to the electronic supplementary material.

Supplementary Material 1

## Data Availability

The data underlying this study are freely accessible to all interested researchers through the UK Biobank database.

## Abbreviations

AA: Aortic aneurysm
AAA: Abdominal aortic aneurysm
TAA: Thoracic aortic aneurysm
CCL: C-C motif chemokine ligand
CCR5: C-C chemokine receptor type-5
MMP9: Matrix metalloproteinase-9
IL-6: Interleukin-6
MMP12: Matrix metallopeptidase-12
CXCL17: C-X-C motif chemokine ligand-17
WFDC2: WAP four-disulfide core domain-2
PLAUR: Plasminogen activator, urokinase receptor
SERPINA1: Serpin family A member-1
SERPINA3: Serpin family A member-3
orm1: Orosomucoid-1
COL18A1: Collagen type XVIII alpha 1 chain
ADM: Adrenomedullin
CST3: Cystatin C
