# Supplementary material for: Sex-specific prediction models for aortic aneurysm integrating traditional clinical risk factors and proteomic profiles: a large-scale prospective study from the UK Biobank
Source: Biol Sex Differ. 2026 May 11;17:128. doi: 10.1186/s13293-026-00913-w (PMC13335366; doi:10.1186/s13293-026-00913-w)
Supplement: Supplementary file 1 — Supplementary Material 1 [file 13293_2026_913_MOESM1_ESM.docx]

**Sex-Specific Prediction Models for Aortic Aneurysm Integrating Traditional Clinical Risk Factors and Proteomic Profiles: A Large- Scale Prospective Study from the UK Biobank**

[Supplementary table 1. The disease corresponds to ICD 10 code. 2](#_Toc226921609)

[Supplementary table 2. Distribution of aortic aneurysm subtypes based on ICD-10 codes in the study cohort. 3](#_Toc226921610)

[Supplementary table 3. Protein missing data imputation status in men. 3](#_Toc226921611)

[Supplementary table 4. Protein missing data imputation status in women. 12](#_Toc226921612)

[Supplementary table 5. Coefficients of candidate proteins for constructing protein risk scores in men and women. 15](#_Toc226921613)

[Supplementary table 6. Proteins Selected by LASSO Cox Regression and Their Corresponding Coefficients in the Sensitivity Analysis Using K-Nearest Neighbors (KNN) Imputation, Stratified by Sex. 16](#_Toc226921614)

[Supplementary table 7. Sensitivity analysis using Fine-Gray subdistribution hazard models to assess the associations between traditional clinical risk factors and incident AA. 16](#_Toc226921615)

[Supplementary table 8. Associations between clinical risk factors and incident AA, AAA, and TAA in male participants. 18](#_Toc226921616)

[Supplementary table 9. Associations between clinical risk factors and incident AA, AAA, and TAA in female participants. 18](#_Toc226921617)

[Supplementary table 10. 5-year,10-year and 15-year PAR of Risk Factors in Men. 19](#_Toc226921618)

[Supplementary table 11. 5-year,10-year and 15-year PAR of Risk Factors in Women. 20](#_Toc226921619)

[Supplementary table 12. Aassociation between proteins and AA in men. 21](#_Toc226921620)

[Supplementary table 13. Aassociation between proteins and AA in women. 29](#_Toc226921621)

[Supplementary table 14. Enrichment analysis results for Reactome pathways in men. 34](#_Toc226921622)

[Supplementary table 15. Enrichment analysis results for Reactome pathways in women. 35](#_Toc226921623)

[Supplementary table 16. Association between Protein Risk Score and Risk of Aortic Aneurysm Using Fine-Gray Competing Risk Models with All-Cause Mortality as the Competing Event. 36](#_Toc226921624)

[Supplementary table 17. Calibration Performance of the Predictive Models in the Independent Validation Cohort. 36](#_Toc226921625)

[Supplementary table 18. Predictive performance of the final model incorporating the protein risk score derived from LASSO regression following KNN imputation of missing protein measurements in internal and external validation cohorts. 36](#_Toc226921626)

# Supplementary table 1. The disease corresponds to ICD 10 code.

| **Disease** | **ICD10** |
| --- | --- |
| **Aortic aneurysm** |  |
| Thoracic aortic aneurysm, ruptured | I71.1 |
| Thoracic aortic aneurysm, without mention of rupture | I71.2 |
| Abdominal aortic aneurysm, ruptured | I71.3 |
| Abdominal aortic aneurysm, without mention of rupture | I71.4 |
| Thoracoabdominal aortic aneurysm, ruptured | I71.5 |
| Thoracoabdominal aortic aneurysm, without mention of rupture | I71.6 |
| Aortic aneurysm of unspecified site, ruptured | I71.8 |
| Aortic aneurysm of unspecified site, without mention of rupture | I71.9 |
| **Valvular heart disease** |  |
| Multiple valve diseases | I08 |
| Nonrheumatic mitral valve disorders | I34 |
| Nonrheumatic aortic valve disorders | I35 |
| Nonrheumatic tricuspid valve disorders | I36 |
| Endocarditis and heart valve disorders in diseases classified elsewhere | I39 |
| **Coronary heart disease** | I20-I25 |
| **Essential (primary) hypertension** | I10 |
| **Disorders of lipoprotein metabolism and other lipidaemias** | E78 |
| **Stroke** | I60-I64 |

# Supplementary table 2. Distribution of aortic aneurysm subtypes based on ICD-10 codes in the study cohort.

| **Subtype** | **ICD-10 Codes** | **Total (n=3,743)** | **Male (n=2,955)** | **Female (n=788)** |
| --- | --- | --- | --- | --- |
| **Abdominal aortic aneurysm (AAA)** | I71.3, I71.4 | 2,221 (59.3%) | 1,877 (63.5%) | 344 (43.6%) |
| --AAA, ruptured | I71.3 | 100 (2.7%) | 86 (2.9%) | 14 (1.8%) |
| --AAA, without ruptured | I71.4 | 2,121 (56.7%) | 1,791 (60.6%) | 330 (41.8%) |
| **Thoracic aortic aneurysm (TAA)** | I71.1, I71.2 | 941 (25.1%) | 648 (21.9%) | 293 (37.1%) |
| --TAA, ruptured | I71.1 | 18 (0.5%) | 7 (0.2%) | 11 (1.4%) |
| --TAA, without ruptured | I71.2 | 923 (24.7%) | 641 (21.7%) | 282 (35.7%) |
| **Thoracoabdominal aortic aneurysm** | I71.5, I71.6 | 32 (0.9%) | 16 (0.5%) | 16 (2.0%) |
| **Aortic aneurysm of unspecified site** | I71.8, I71.9 | 549 (14.7%) | 414 (14.0%) | 135 (17.1%) |

# Supplementary table 3. Protein missing data imputation status in men.

|  | **Training set** | | **Internal validation set** | | **External validation set** | |
| --- | --- | --- | --- | --- | --- | --- |
| **Protein** | **Fill value** | **Fill the quantity** | **Fill value** | **Fill the quantity** | **Fill value** | **Fill the quantity** |
| mmp12 | -2.5077 | 396 | -2.6317 | 158 | -2.3687 | 74 |
| cxcl17 | -1.9946 | 239 | -2.9865 | 111 | -1.5659 | 49 |
| il6 | -2.1633 | 456 | -2.2493 | 194 | -1.9044 | 109 |
| ceacam5 | -2.3582 | 869 | -3.1462 | 369 | -2.0206 | 225 |
| s100a12 | -3.1304 | 562 | -2.447 | 254 | -2.9732 | 159 |
| ctsl | -1.0551 | 380 | -0.9462 | 168 | -0.9586 | 106 |
| il7r | -2.8856 | 390 | -2.4065 | 181 | -2.2953 | 91 |
| egfr | -1.0019 | 221 | -1.0954 | 101 | -0.7301 | 56 |
| tnfrsf11b | -1.2224 | 396 | -1.4131 | 208 | -1.312 | 103 |
| dpp6 | -1.6427 | 456 | -1.5784 | 194 | -1.5637 | 109 |
| enpp5 | -4.988 | 339 | -4.5315 | 171 | -4.5802 | 83 |
| klk4 | -2.5395 | 280 | -2.8235 | 121 | -2.5928 | 58 |
| mln | -3.9912 | 393 | -3.7976 | 190 | -3.606 | 99 |
| igfbpl1 | -1.7509 | 380 | -1.5202 | 168 | -1.3485 | 106 |
| ntprobnp | -4.582 | 380 | -4.1873 | 168 | -3.3651 | 106 |
| gcnt1 | -1.5967 | 456 | -1.9194 | 194 | -1.8024 | 109 |
| sorcs2 | -2.1289 | 695 | -2.5075 | 311 | -1.7674 | 181 |
| st8sia1 | -1.3426 | 2593 | -1.2531 | 1006 | -1.271 | 588 |
| tnfsf13b | -1.6342 | 238 | -1.9124 | 111 | -1.6144 | 67 |
| edar | -3.3302 | 393 | -3.1849 | 190 | -2.792 | 99 |
| ctsv | -2.5932 | 348 | -1.5878 | 123 | -2.0946 | 74 |
| cd300lg | -1.8969 | 390 | -1.8561 | 181 | -2.0031 | 91 |
| lpa | -5.5926 | 2552 | -5.1691 | 924 | -5.4349 | 551 |
| ca14 | -1.8093 | 280 | -1.7748 | 121 | -1.8644 | 58 |
| cfc1 | -2.8943 | 695 | -2.5666 | 311 | -1.5998 | 181 |
| smad3 | -2.4795 | 2327 | -1.4266 | 866 | -1.7735 | 496 |
| rgma | -1.7228 | 266 | -1.6413 | 133 | -1.6676 | 65 |
| ccl21 | -1.9314 | 339 | -1.4996 | 171 | -1.4512 | 83 |
| anxa10 | -2.7709 | 677 | -3.0324 | 315 | -4.9929 | 166 |
| vstm2l | -2.4619 | 605 | -2.721 | 287 | -2.4518 | 166 |
| saa4 | -0.607 | 2269 | -0.7302 | 840 | -0.8768 | 488 |
| boc | -1.2316 | 605 | -1.9069 | 287 | -0.8022 | 166 |
| adgrg1 | -1.8076 | 456 | -1.3419 | 194 | -1.4769 | 109 |
| wfdc2 | -2.7379 | 1078 | -- | -- | -- | -- |
| npc2 | -2.1665 | 2452 | -- | -- | -- | -- |
| plaur | -1.7753 | 468 | -- | -- | -- | -- |
| prss8 | -1.5057 | 446 | -- | -- | -- | -- |
| ca6 | -3.6196 | 430 | -- | -- | -- | -- |
| hgf | -1.3004 | 239 | -- | -- | -- | -- |
| ccl7 | -1.6964 | 393 | -- | -- | -- | -- |
| cst3 | -1.9109 | 354 | -- | -- | -- | -- |
| lamp3 | -2.5566 | 260 | -- | -- | -- | -- |
| msln | -1.9968 | 469 | -- | -- | -- | -- |
| alpp | -4.184 | 456 | -- | -- | -- | -- |
| spp1 | -2.5596 | 241 | -- | -- | -- | -- |
| orm1 | -0.9177 | 2315 | -- | -- | -- | -- |
| tnfrsf10b | -1.3166 | 500 | -- | -- | -- | -- |
| col18a1 | -1.3378 | 324 | -- | -- | -- | -- |
| adm | -2.6537 | 280 | -- | -- | -- | -- |
| ifi30 | -2.7642 | 2551 | -- | -- | -- | -- |
| cd83 | -1.5976 | 393 | -- | -- | -- | -- |
| igfbp4 | -2.7354 | 538 | -- | -- | -- | -- |
| rnase1 | -1.1764 | 2283 | -- | -- | -- | -- |
| rnase6 | -1.9518 | 2363 | -- | -- | -- | -- |
| pgf | -1.4907 | 239 | -- | -- | -- | -- |
| gdf15 | -1.9474 | 221 | -- | -- | -- | -- |
| ccl16 | -3.8006 | 221 | -- | -- | -- | -- |
| trem2 | -2.9318 | 396 | -- | -- | -- | -- |
| shisa5 | -1.3202 | 2273 | -- | -- | -- | -- |
| ggh | -2.4517 | 625 | -- | -- | -- | -- |
| prap1 | -2.6878 | 2273 | -- | -- | -- | -- |
| tnfrsf9 | -1.6796 | 500 | -- | -- | -- | -- |
| acvrl1 | -1.0292 | 531 | -- | -- | -- | -- |
| ocln | -1.8178 | 2311 | -- | -- | -- | -- |
| asgr1 | -1.4819 | 500 | -- | -- | -- | -- |
| rarres2 | -2.0829 | 310 | -- | -- | -- | -- |
| edn1 | -1.6376 | 2591 | -- | -- | -- | -- |
| vwc2 | -3.8024 | 552 | -- | -- | -- | -- |
| f9 | -2.2564 | 350 | -- | -- | -- | -- |
| il2ra | -1.367 | 457 | -- | -- | -- | -- |
| scarb2 | -1.8157 | 750 | -- | -- | -- | -- |
| rnase4 | -3.1814 | 2649 | -- | -- | -- | -- |
| efna4 | -1.4247 | 531 | -- | -- | -- | -- |
| xcl1 | -1.7006 | 280 | -- | -- | -- | -- |
| b2m | -1.1967 | 2269 | -- | -- | -- | -- |
| vsig4 | -5.8124 | 405 | -- | -- | -- | -- |
| vsig2 | -2.3544 | 2327 | -- | -- | -- | -- |
| lilrb4 | -2.0406 | 588 | -- | -- | -- | -- |
| tnfrsf10a | -1.5135 | 500 | -- | -- | -- | -- |
| lgals9 | -1.6688 | 396 | -- | -- | -- | -- |
| igfbp7 | -1.4711 | 221 | -- | -- | -- | -- |
| sftpa2 | -1.6775 | 695 | -- | -- | -- | -- |
| tnfrsf1b | -1.3445 | 390 | -- | -- | -- | -- |
| st6gal1 | -1.7618 | 221 | -- | -- | -- | -- |
| tnfrsf1a | -1.1586 | 390 | -- | -- | -- | -- |
| havcr2 | -1.8897 | 390 | -- | -- | -- | -- |
| tff2 | -4.5195 | 357 | -- | -- | -- | -- |
| lair1 | -2.057 | 396 | -- | -- | -- | -- |
| colec12 | -1.7221 | 396 | -- | -- | -- | -- |
| ceacam6 | -1.396 | 2273 | -- | -- | -- | -- |
| hla_e | -1.4996 | 393 | -- | -- | -- | -- |
| msr1 | -6.8932 | 266 | -- | -- | -- | -- |
| plat | -2.9049 | 262 | -- | -- | -- | -- |
| tff3 | -1.2712 | 391 | -- | -- | -- | -- |
| col6a3 | -2.2813 | 221 | -- | -- | -- | -- |
| igdcc4 | -3.9437 | 2273 | -- | -- | -- | -- |
| smoc1 | -1.9953 | 280 | -- | -- | -- | -- |
| cxcl16 | -1.4138 | 238 | -- | -- | -- | -- |
| tnfrsf6b | -2.4366 | 500 | -- | -- | -- | -- |
| clec5a | -1.4486 | 420 | -- | -- | -- | -- |
| cd80 | -2.3302 | 2289 | -- | -- | -- | -- |
| fam20a | -1.7485 | 2289 | -- | -- | -- | -- |
| cfi | -1.1093 | 2269 | -- | -- | -- | -- |
| mmp7 | -4.4838 | 632 | -- | -- | -- | -- |
| crip2 | -1.765 | 500 | -- | -- | -- | -- |
| rnf149 | -1.1937 | 2591 | -- | -- | -- | -- |
| cd74 | -2.063 | 266 | -- | -- | -- | -- |
| spink1 | -2.6286 | 390 | -- | -- | -- | -- |
| rnaset2 | -1.5927 | 283 | -- | -- | -- | -- |
| pigr | -1.8231 | 566 | -- | -- | -- | -- |
| angpt2 | -1.5194 | 280 | -- | -- | -- | -- |
| tnf | -1.27 | 624 | -- | -- | -- | -- |
| cd27 | -3.3486 | 370 | -- | -- | -- | -- |
| sel1l | -0.9899 | 2591 | -- | -- | -- | -- |
| inhbb | -2.391 | 2559 | -- | -- | -- | -- |
| eda2r | -6.038 | 280 | -- | -- | -- | -- |
| gpr37 | -2.519 | 605 | -- | -- | -- | -- |
| tnfrsf13b | -2.9729 | 377 | -- | -- | -- | -- |
| reg1b | -1.8383 | 221 | -- | -- | -- | -- |
| clec4d | -2.8174 | 393 | -- | -- | -- | -- |
| ckap4 | -1.2342 | 299 | -- | -- | -- | -- |
| ptgds | -1.3504 | 490 | -- | -- | -- | -- |
| cfb | -2.1113 | 2444 | -- | -- | -- | -- |
| psap | -1.302 | 2352 | -- | -- | -- | -- |
| furin | -3.5107 | 370 | -- | -- | -- | -- |
| relt | -1.1307 | 390 | -- | -- | -- | -- |
| cd300lf | -3.5289 | 280 | -- | -- | -- | -- |
| bsg | -3.977 | 260 | -- | -- | -- | -- |
| fgf23 | -2.6349 | 311 | -- | -- | -- | -- |
| gm2a | -1.3109 | 2526 | -- | -- | -- | -- |
| gchfr | -3.8147 | 2828 | -- | -- | -- | -- |
| cpxm2 | -1.2632 | 2289 | -- | -- | -- | -- |
| havcr1 | -3.2802 | 306 | -- | -- | -- | -- |
| cdcp1 | -1.7871 | 500 | -- | -- | -- | -- |
| il10rb | -1.8325 | 339 | -- | -- | -- | -- |
| serpina1 | -0.7461 | 2454 | -- | -- | -- | -- |
| cfh | -1.253 | 2903 | -- | -- | -- | -- |
| lrg1 | -2.1196 | 2269 | -- | -- | -- | -- |
| siglec7 | -1.3737 | 380 | -- | -- | -- | -- |
| sit1 | -2.309 | 588 | -- | -- | -- | -- |
| ang | -1.8198 | 413 | -- | -- | -- | -- |
| csf1 | -1.1059 | 339 | -- | -- | -- | -- |
| fgfbp1 | -4.2567 | 370 | -- | -- | -- | -- |
| ctsd | -1.8969 | 1259 | -- | -- | -- | -- |
| spink2 | -1.1382 | 2559 | -- | -- | -- | -- |
| cfd | -1.1247 | 2269 | -- | -- | -- | -- |
| guk1 | -1.3977 | 2668 | -- | -- | -- | -- |
| gfra1 | -1.9372 | 301 | -- | -- | -- | -- |
| lrrn1 | -2.0465 | 588 | -- | -- | -- | -- |
| il18bp | -1.4978 | 324 | -- | -- | -- | -- |
| icam1 | -1.7493 | 448 | -- | -- | -- | -- |
| pon3 | -7.231 | 339 | -- | -- | -- | -- |
| ccl22 | -2.9007 | 494 | -- | -- | -- | -- |
| agrn | -1.7068 | 339 | -- | -- | -- | -- |
| chchd10 | -3.8441 | 2315 | -- | -- | -- | -- |
| cd59 | -1.0095 | 884 | -- | -- | -- | -- |
| prss2 | -3.6708 | 324 | -- | -- | -- | -- |
| il1rn | -1.7724 | 423 | -- | -- | -- | -- |
| serpinf1 | -1.3801 | 2412 | -- | -- | -- | -- |
| apom | -2.0524 | 350 | -- | -- | -- | -- |
| prune2 | -1.0048 | 2493 | -- | -- | -- | -- |
| itih3 | -2.3862 | 324 | -- | -- | -- | -- |
| edil3 | -1.5748 | 605 | -- | -- | -- | -- |
| timp1 | -1.3262 | 513 | -- | -- | -- | -- |
| fstl3 | -1.6634 | 423 | -- | -- | -- | -- |
| reg1a | -1.574 | 483 | -- | -- | -- | -- |
| pga4 | -5.7326 | 2283 | -- | -- | -- | -- |
| il22 | -2.1456 | 2327 | -- | -- | -- | -- |
| septin3 | -2.8624 | 2311 | -- | -- | -- | -- |
| cdh2 | -2.0279 | 380 | -- | -- | -- | -- |
| lepr | -1.6527 | 380 | -- | -- | -- | -- |
| rspo1 | -2.0706 | 500 | -- | -- | -- | -- |
| ulbp2 | -1.9224 | 500 | -- | -- | -- | -- |
| siglec1 | -2.5098 | 339 | -- | -- | -- | -- |
| capg | -5.1802 | 487 | -- | -- | -- | -- |
| rtn4r | -1.6562 | 280 | -- | -- | -- | -- |
| pik3ip1 | -1.4887 | 390 | -- | -- | -- | -- |
| mzb1 | -4.5931 | 359 | -- | -- | -- | -- |
| reg3a | -2.085 | 221 | -- | -- | -- | -- |
| creld1 | -1.7879 | 2559 | -- | -- | -- | -- |
| dner | -1.6705 | 416 | -- | -- | -- | -- |
| penk | -1.2369 | 2559 | -- | -- | -- | -- |
| pzp | -2.0791 | 2269 | -- | -- | -- | -- |
| fut3_fut5 | -2.2665 | 266 | -- | -- | -- | -- |
| hspg2 | -1.3608 | 461 | -- | -- | -- | -- |
| siglec8 | -2.3805 | 2591 | -- | -- | -- | -- |
| tff1 | -2.2794 | 453 | -- | -- | -- | -- |
| tmprss11d | -3.7038 | 2322 | -- | -- | -- | -- |
| fabp3 | -1.86 | 2289 | -- | -- | -- | -- |
| mfap5 | -2.8237 | 221 | -- | -- | -- | -- |
| eln | -2.442 | 2289 | -- | -- | -- | -- |
| ambp | -1.5176 | 306 | -- | -- | -- | -- |
| lcn2 | -2.0122 | 789 | -- | -- | -- | -- |
| sftpd | -3.0112 | 380 | -- | -- | -- | -- |
| chga | -2.5615 | 2273 | -- | -- | -- | -- |
| cd70 | -2.1385 | 393 | -- | -- | -- | -- |
| met | -1.0668 | 363 | -- | -- | -- | -- |
| oscar | -2.1854 | 377 | -- | -- | -- | -- |
| ogn | -2.4615 | 390 | -- | -- | -- | -- |
| icam5 | -2.2135 | 380 | -- | -- | -- | -- |
| aldh3a1 | -2.3245 | 407 | -- | -- | -- | -- |
| il18r1 | -1.4061 | 239 | -- | -- | -- | -- |
| lta4h | -2.2612 | 280 | -- | -- | -- | -- |
| ncan | -2.8922 | 266 | -- | -- | -- | -- |
| npl | -3.6436 | 2289 | -- | -- | -- | -- |
| pla2g15 | -3.9542 | 370 | -- | -- | -- | -- |
| cd300e | -2.6605 | 311 | -- | -- | -- | -- |
| ccl14 | -2.3655 | 413 | -- | -- | -- | -- |
| fga | -1.3584 | 2511 | -- | -- | -- | -- |
| agrp | -2.6742 | 239 | -- | -- | -- | -- |
| cd207 | -3.821 | 663 | -- | -- | -- | -- |
| cfhr5 | -4.0177 | 2425 | -- | -- | -- | -- |
| gfral | -2.6766 | 2620 | -- | -- | -- | -- |
| iglc2 | -0.8442 | 2281 | -- | -- | -- | -- |
| lmnb2 | -2.1122 | 2315 | -- | -- | -- | -- |
| gipc2 | -1.1701 | 2315 | -- | -- | -- | -- |
| lgals4 | -2.0709 | 396 | -- | -- | -- | -- |
| pag1 | -2.0082 | 605 | -- | -- | -- | -- |
| cd4 | -1.2663 | 411 | -- | -- | -- | -- |
| sema3f | -1.0411 | 380 | -- | -- | -- | -- |
| rab6a | -3.2605 | 393 | -- | -- | -- | -- |
| fabp4 | -2.7214 | 221 | -- | -- | -- | -- |
| ryr1 | -1.2528 | 2668 | -- | -- | -- | -- |
| tnfrsf11a | -1.7106 | 239 | -- | -- | -- | -- |
| osm | -2.956 | 455 | -- | -- | -- | -- |
| itgb5 | -3.4663 | 370 | -- | -- | -- | -- |
| defa1_defa1b | -2.5379 | 655 | -- | -- | -- | -- |
| tnfrsf4 | -1.7889 | 260 | -- | -- | -- | -- |
| cstb | -2.0216 | 558 | -- | -- | -- | -- |
| bpifb1 | -7.1819 | 404 | -- | -- | -- | -- |
| rbp5 | -2.2082 | 456 | -- | -- | -- | -- |
| retn | -3.0189 | 433 | -- | -- | -- | -- |
| ntrk3 | -1.4407 | 266 | -- | -- | -- | -- |
| nppb | -8.4628 | 605 | -- | -- | -- | -- |
| pilra | -3.8647 | 390 | -- | -- | -- | -- |
| commd9 | -1.9432 | 2327 | -- | -- | -- | -- |
| dpp4 | -2.2268 | 619 | -- | -- | -- | -- |
| ada2 | -2.2886 | 468 | -- | -- | -- | -- |
| krt19 | -2.125 | 407 | -- | -- | -- | -- |
| chrdl1 | -2.9688 | 357 | -- | -- | -- | -- |
| cdan1 | -1.2143 | 2315 | -- | -- | -- | -- |
| cntn1 | -4.2507 | 221 | -- | -- | -- | -- |
| adam12 | -1.2381 | 2593 | -- | -- | -- | -- |
| ccl23 | -2.8846 | 339 | -- | -- | -- | -- |
| pilrb | -2.3658 | 380 | -- | -- | -- | -- |
| cntn5 | -1.6587 | 353 | -- | -- | -- | -- |
| lilra5 | -1.8695 | 404 | -- | -- | -- | -- |
| fabp1 | -3.483 | 396 | -- | -- | -- | -- |
| tgfa | -2.1732 | 393 | -- | -- | -- | -- |
| fap | -1.8158 | 221 | -- | -- | -- | -- |
| il16 | -2.5309 | 239 | -- | -- | -- | -- |
| pon1 | -3.1188 | 2283 | -- | -- | -- | -- |
| efemp1 | -1.5799 | 340 | -- | -- | -- | -- |
| dsc2 | -1.6853 | 545 | -- | -- | -- | -- |
| pamr1 | -1.5772 | 390 | -- | -- | -- | -- |
| msmb | -2.7854 | 386 | -- | -- | -- | -- |
| spon2 | -1.702 | 448 | -- | -- | -- | -- |
| serpind1 | -1.7861 | 2291 | -- | -- | -- | -- |
| hjv | -2.242 | 2311 | -- | -- | -- | -- |
| gast | -2.0524 | 2315 | -- | -- | -- | -- |
| nectin2 | -1.1307 | 404 | -- | -- | -- | -- |
| col15a1 | -1.9272 | 2273 | -- | -- | -- | -- |
| tgfbr2 | -2.8316 | 370 | -- | -- | -- | -- |
| sfrp1 | -2.4126 | 559 | -- | -- | -- | -- |
| clec14a | -1.4394 | 390 | -- | -- | -- | -- |
| itih4 | -1.3846 | 2269 | -- | -- | -- | -- |
| tnfrsf17 | -4.4331 | 2559 | -- | -- | -- | -- |
| pltp | -2.4048 | 262 | -- | -- | -- | -- |
| klk14 | -2.2624 | 280 | -- | -- | -- | -- |
| nos3 | -1.888 | 677 | -- | -- | -- | -- |
| scrg1 | -1.503 | 2289 | -- | -- | -- | -- |
| serpina3 | -0.8705 | 2304 | -- | -- | -- | -- |
| cd7 | -2.1518 | 2614 | -- | -- | -- | -- |
| igfbp6 | -1.7435 | 442 | -- | -- | -- | -- |
| mad1l1 | -1.3433 | 531 | -- | -- | -- | -- |
| epha1 | -2.4804 | 366 | -- | -- | -- | -- |
| efna1 | -1.3216 | 408 | -- | -- | -- | -- |
| adamts15 | -3.9393 | 456 | -- | -- | -- | -- |
| ddc | -2.5616 | 404 | -- | -- | -- | -- |
| lilrb2 | -2.2894 | 282 | -- | -- | -- | -- |
| cd300a | -1.364 | 2559 | -- | -- | -- | -- |
| ncam1 | -1.6809 | 324 | -- | -- | -- | -- |
| cd5 | -2.3498 | 306 | -- | -- | -- | -- |
| rnase10 | -1.6764 | 2591 | -- | -- | -- | -- |
| ahnak | -2.3953 | 2591 | -- | -- | -- | -- |
| itga11 | -2.0388 | 393 | -- | -- | -- | -- |
| itgav | -0.9434 | 280 | -- | -- | -- | -- |
| bcan | -2.034 | 500 | -- | -- | -- | -- |
| tgfbr1 | -0.7636 | 2593 | -- | -- | -- | -- |
| lrig1 | -2.0258 | 327 | -- | -- | -- | -- |
| mmp9 | -2.9934 | 412 | -- | -- | -- | -- |
| cd38 | -1.4281 | 456 | -- | -- | -- | -- |
| a1bg | -1.0273 | 2269 | -- | -- | -- | -- |
| ccn1 | -3.578 | 482 | -- | -- | -- | -- |
| ptp4a3 | -1.4858 | 2593 | -- | -- | -- | -- |
| serpina11 | -2.7547 | 221 | -- | -- | -- | -- |
| clgn | -1.7118 | 2586 | -- | -- | -- | -- |
| ctss | -1.0748 | 3150 | -- | -- | -- | -- |
| yap1 | -3.2869 | 2315 | -- | -- | -- | -- |
| ermap | -1.7788 | 2593 | -- | -- | -- | -- |
| cd274 | -1.0519 | 500 | -- | -- | -- | -- |
| scg2 | -1.4078 | 339 | -- | -- | -- | -- |
| nt5c1a | -2.0355 | 2315 | -- | -- | -- | -- |
| pglyrp1 | -2.0019 | 282 | -- | -- | -- | -- |
| tnfrsf12a | -2.3887 | 370 | -- | -- | -- | -- |
| c1qa | -1.1851 | 306 | -- | -- | -- | -- |
| chi3l1 | -2.6089 | 644 | -- | -- | -- | -- |
| skap1 | -2.9451 | 521 | -- | -- | -- | -- |
| ccl27 | -8.3304 | 380 | -- | -- | -- | -- |
| polr2f | -1.1471 | 456 | -- | -- | -- | -- |
| clec6a | -3.4623 | 695 | -- | -- | -- | -- |
| adam8 | -4.5702 | 285 | -- | -- | -- | -- |
| wfikkn2 | -5.9478 | 377 | -- | -- | -- | -- |
| npdc1 | -1.2207 | 380 | -- | -- | -- | -- |
| prtn3 | -1.94 | 719 | -- | -- | -- | -- |
| lgals3bp | -1.1914 | 2269 | -- | -- | -- | -- |
| bambi | -1.2879 | 695 | -- | -- | -- | -- |
| clec12a | -1.1937 | 2593 | -- | -- | -- | -- |
| ephb4 | -1.1632 | 221 | -- | -- | -- | -- |
| apoa1 | -1.3824 | 2269 | -- | -- | -- | -- |
| nt5c3a | -4.2027 | 416 | -- | -- | -- | -- |
| ptprb | -2.1723 | 2289 | -- | -- | -- | -- |
| btn3a2 | -2.0274 | 393 | -- | -- | -- | -- |
| tnfsf13 | -1.6053 | 339 | -- | -- | -- | -- |
| fas | -1.2139 | 262 | -- | -- | -- | -- |
| krt8 | -2.1942 | 2642 | -- | -- | -- | -- |
| stc1 | -1.5822 | 266 | -- | -- | -- | -- |
| sema3g | -1.514 | 2559 | -- | -- | -- | -- |
| apcs | -2.7227 | 2375 | -- | -- | -- | -- |
| hip1r | -2.2283 | 2342 | -- | -- | -- | -- |
| spon1 | -1.9944 | 377 | -- | -- | -- | -- |
| palm2 | -2.1028 | 2315 | -- | -- | -- | -- |
| rida | -1.7549 | 2559 | -- | -- | -- | -- |
| gas6 | -1.4005 | 299 | -- | -- | -- | -- |
| ccl11 | -5.0122 | 239 | -- | -- | -- | -- |
| vwa1 | -3.1978 | 301 | -- | -- | -- | -- |
| cxcl9 | -1.8635 | 339 | -- | -- | -- | -- |
| mxra8 | -1.5019 | 2559 | -- | -- | -- | -- |
| lrrc25 | -1.6261 | 456 | -- | -- | -- | -- |
| cxcl13 | -1.9145 | 266 | -- | -- | -- | -- |
| adamtsl2 | -1.9212 | 2273 | -- | -- | -- | -- |
| lrrc38 | -1.7257 | 2586 | -- | -- | -- | -- |
| fgf21 | -4.1925 | 301 | -- | -- | -- | -- |
| efcab14 | -2.4091 | 2318 | -- | -- | -- | -- |
| gpc5 | -2.8343 | 500 | -- | -- | -- | -- |
| idua | -3.1409 | 280 | -- | -- | -- | -- |
| scn4b | -2.0065 | 2591 | -- | -- | -- | -- |
| pth | -3.5075 | 2586 | -- | -- | -- | -- |
| clec10a | -1.897 | 500 | -- | -- | -- | -- |
| nmnat1 | -2.6493 | 500 | -- | -- | -- | -- |
| adamts1 | -1.4959 | 2593 | -- | -- | -- | -- |
| asgr2 | -1.6006 | 2283 | -- | -- | -- | -- |
| gal | -2.4385 | 339 | -- | -- | -- | -- |
| ism1 | -1.9866 | 393 | -- | -- | -- | -- |
| pi3 | -1.5037 | 545 | -- | -- | -- | -- |
| b4gat1 | -1.6805 | 390 | -- | -- | -- | -- |
| bmper | -1.0626 | 2559 | -- | -- | -- | -- |
| layn | -1.8338 | 521 | -- | -- | -- | -- |
| aplp1 | -2.7851 | 380 | -- | -- | -- | -- |
| vegfa | -1.6696 | 280 | -- | -- | -- | -- |
| prg2 | -2.1297 | 2273 | -- | -- | -- | -- |
| itgam | -1.4182 | 390 | -- | -- | -- | -- |
| klrk1 | -3.3039 | 2315 | -- | -- | -- | -- |
| ment | -2.1054 | 2559 | -- | -- | -- | -- |
| ctag1a_ctag1b | -3.3645 | 2517 | -- | -- | -- | -- |
| itgb1 | -1.4216 | 386 | -- | -- | -- | -- |
| notch2 | -1.3471 | 2273 | -- | -- | -- | -- |
| ltbp2 | -3.8575 | 221 | -- | -- | -- | -- |
| hdac9 | -2.3516 | 2642 | -- | -- | -- | -- |
| lect2 | -2.9267 | 2289 | -- | -- | -- | -- |
| mcam | -1.7706 | 324 | -- | -- | -- | -- |
| adamts8 | -4.0692 | 370 | -- | -- | -- | -- |
| ptprn2 | -1.3925 | 549 | -- | -- | -- | -- |
| ccl3 | -1.935 | 393 | -- | -- | -- | -- |
| tfrc | -2.1631 | 330 | -- | -- | -- | -- |
| ceacam8 | -2.755 | 605 | -- | -- | -- | -- |
| cd72 | -1.889 | 2559 | -- | -- | -- | -- |
| fcamr | -2.957 | 2289 | -- | -- | -- | -- |
| pnliprp1 | -5.5885 | 2315 | -- | -- | -- | -- |
| ccl15 | -1.3278 | 221 | -- | -- | -- | -- |
| osmr | -1.0288 | 310 | -- | -- | -- | -- |
| c1s | -1.8917 | 2269 | -- | -- | -- | -- |
| tab2 | -4.3596 | 2493 | -- | -- | -- | -- |
| aspscr1 | -3.0583 | 2311 | -- | -- | -- | -- |
| csnk2a1 | -1.9813 | 2311 | -- | -- | -- | -- |
| cyp24a1 | -2.46 | 2668 | -- | -- | -- | -- |
| cd34 | -1.3864 | 311 | -- | -- | -- | -- |
| kitlg | -2.6749 | 380 | -- | -- | -- | -- |
| umod | -3.9859 | 271 | -- | -- | -- | -- |
| ctsz | -3.1533 | 362 | -- | -- | -- | -- |
| nell1 | -3.7469 | 370 | -- | -- | -- | -- |
| mamdc2 | -1.5173 | 2289 | -- | -- | -- | -- |
| mpo | -3.7058 | 390 | -- | -- | -- | -- |
| c1r | -1.0566 | 2269 | -- | -- | -- | -- |
| slc4a1 | -1.9751 | 2273 | -- | -- | -- | -- |

Fill value: minima of columns; Fill the quantity: number of participants with missing protein expression values; The validation set only interpolated the proteins screened by LASSO.

# Supplementary table 4. Protein missing data imputation status in women.

|  | **Training set** | | **Internal validation set** | | **External validation set** | |
| --- | --- | --- | --- | --- | --- | --- |
| **Protein** | **Fill value** | **Fill the quantity** | **Fill value** | **Fill the quantity** | **Fill value** | **Fill the quantity** |
| mmp12 | -2.814 | 464 | -2.6914 | 193 | -2.156 | 85 |
| cxcl17 | -3.4149 | 287 | -2.4195 | 122 | -2.251 | 72 |
| tnfrsf10b | -1.4737 | 576 | -1.6764 | 237 | -1.603 | 121 |
| plaur | -1.7963 | 539 | -1.5309 | 230 | -1.3127 | 142 |
| alpp | -4.4371 | 520 | -4.9495 | 226 | -3.912 | 116 |
| il6 | -3.419 | 520 | -3.0096 | 226 | -1.8736 | 116 |
| msln | -1.9606 | 545 | -1.8387 | 237 | -1.841 | 120 |
| ntprobnp | -3.5254 | 455 | -3.8755 | 185 | -3.8987 | 111 |
| gal | -2.8586 | 366 | -2.7992 | 157 | -2.4902 | 92 |
| cbln4 | -2.6068 | 330 | -2.4295 | 134 | -2.4789 | 64 |
| ret | -2.2706 | 520 | -1.9213 | 226 | -1.8148 | 116 |
| ncan | -2.6988 | 324 | -2.2224 | 141 | -3.2461 | 58 |
| cst7 | -2.4431 | 416 | -2.2147 | 179 | -2.0816 | 106 |
| wfdc2 | -3.2549 | 1268 | -- | -- | -- | -- |
| lamp3 | -2.2591 | 308 | -- | -- | -- | -- |
| gdf15 | -2.0238 | 253 | -- | -- | -- | -- |
| pgf | -1.5459 | 287 | -- | -- | -- | -- |
| tnfsf13b | -1.8166 | 275 | -- | -- | -- | -- |
| igfbp4 | -2.0343 | 672 | -- | -- | -- | -- |
| acvrl1 | -0.9993 | 598 | -- | -- | -- | -- |
| pigr | -2.0236 | 661 | -- | -- | -- | -- |
| sftpa2 | -1.7334 | 835 | -- | -- | -- | -- |
| il19 | -3.4318 | 832 | -- | -- | -- | -- |
| tnfrsf10a | -1.6554 | 576 | -- | -- | -- | -- |
| agrn | -3.2076 | 366 | -- | -- | -- | -- |
| eda2r | -2.0064 | 330 | -- | -- | -- | -- |
| clec5a | -1.5866 | 504 | -- | -- | -- | -- |
| prss8 | -1.9272 | 504 | -- | -- | -- | -- |
| cst3 | -1.6736 | 390 | -- | -- | -- | -- |
| tnfrsf1a | -1.2125 | 465 | -- | -- | -- | -- |
| efna4 | -1.6955 | 598 | -- | -- | -- | -- |
| il15 | -1.7701 | 459 | -- | -- | -- | -- |
| cd83 | -1.5488 | 459 | -- | -- | -- | -- |
| col6a3 | -1.8101 | 253 | -- | -- | -- | -- |
| klk13 | -2.1743 | 756 | -- | -- | -- | -- |
| havcr2 | -3.3394 | 465 | -- | -- | -- | -- |
| vsig4 | -3.4261 | 489 | -- | -- | -- | -- |
| lrg1 | -2.1932 | 2621 | -- | -- | -- | -- |
| tnfrsf1b | -1.5299 | 465 | -- | -- | -- | -- |
| rnase1 | -1.2899 | 2653 | -- | -- | -- | -- |
| ccl3 | -1.9002 | 459 | -- | -- | -- | -- |
| rab6a | -2.8272 | 459 | -- | -- | -- | -- |
| lilrb4 | -2.1644 | 747 | -- | -- | -- | -- |
| scarb2 | -1.8851 | 859 | -- | -- | -- | -- |
| spink1 | -2.3462 | 465 | -- | -- | -- | -- |
| lgals4 | -2.0733 | 434 | -- | -- | -- | -- |
| colec12 | -1.8331 | 434 | -- | -- | -- | -- |
| tnfrsf11a | -1.5916 | 287 | -- | -- | -- | -- |
| tgfa | -1.5938 | 459 | -- | -- | -- | -- |
| tnfrsf6b | -2.4657 | 576 | -- | -- | -- | -- |
| serpina3 | -1.0883 | 2689 | -- | -- | -- | -- |
| adm | -3.8369 | 330 | -- | -- | -- | -- |
| tafa5 | -1.6802 | 520 | -- | -- | -- | -- |
| fgf23 | -2.5722 | 362 | -- | -- | -- | -- |
| ltbr | -1.5336 | 506 | -- | -- | -- | -- |
| il4r | -1.7229 | 459 | -- | -- | -- | -- |
| pik3ip1 | -1.5476 | 465 | -- | -- | -- | -- |
| rbp2 | -2.7605 | 520 | -- | -- | -- | -- |
| msr1 | -5.9605 | 324 | -- | -- | -- | -- |
| orm1 | -0.8855 | 2673 | -- | -- | -- | -- |
| cdcp1 | -1.7692 | 576 | -- | -- | -- | -- |
| spp1 | -4.0444 | 278 | -- | -- | -- | -- |
| ckap4 | -1.1878 | 358 | -- | -- | -- | -- |
| vwc2 | -2.6182 | 623 | -- | -- | -- | -- |
| nectin2 | -1.3783 | 490 | -- | -- | -- | -- |
| lgals9 | -1.7282 | 442 | -- | -- | -- | -- |
| tff2 | -4.6728 | 392 | -- | -- | -- | -- |
| fabp1 | -4.1823 | 434 | -- | -- | -- | -- |
| col18a1 | -1.6336 | 383 | -- | -- | -- | -- |
| igfbp7 | -1.9423 | 253 | -- | -- | -- | -- |
| nppb | -7.8439 | 764 | -- | -- | -- | -- |
| icam5 | -2.2037 | 455 | -- | -- | -- | -- |
| relt | -1.3193 | 465 | -- | -- | -- | -- |
| cd74 | -1.9328 | 324 | -- | -- | -- | -- |
| fstl3 | -1.657 | 460 | -- | -- | -- | -- |
| efna1 | -1.1443 | 487 | -- | -- | -- | -- |
| il15ra | -1.9607 | 747 | -- | -- | -- | -- |
| crhbp | -2.2183 | 434 | -- | -- | -- | -- |
| siglec1 | -5.2034 | 366 | -- | -- | -- | -- |
| clec10a | -2.0666 | 576 | -- | -- | -- | -- |
| cd27 | -3.0943 | 398 | -- | -- | -- | -- |
| epha2 | -1.3621 | 1037 | -- | -- | -- | -- |
| pi3 | -2.1915 | 648 | -- | -- | -- | -- |
| ccl7 | -2.1835 | 459 | -- | -- | -- | -- |
| inhbb | -2.2257 | 2957 | -- | -- | -- | -- |
| csf1 | -1.4312 | 366 | -- | -- | -- | -- |
| il2ra | -2.1989 | 567 | -- | -- | -- | -- |
| cgref1 | -1.9349 | 455 | -- | -- | -- | -- |
| rnase6 | -1.2531 | 2757 | -- | -- | -- | -- |
| trem2 | -4.1024 | 434 | -- | -- | -- | -- |
| clec14a | -1.5287 | 465 | -- | -- | -- | -- |
| areg | -1.8321 | 520 | -- | -- | -- | -- |
| tnr | -3.1926 | 576 | -- | -- | -- | -- |
| ccn3 | -3.1785 | 271 | -- | -- | -- | -- |
| reg4 | -6.5971 | 408 | -- | -- | -- | -- |
| il18bp | -1.7738 | 363 | -- | -- | -- | -- |
| ephb4 | -1.2675 | 253 | -- | -- | -- | -- |
| krt19 | -2.3103 | 475 | -- | -- | -- | -- |
| defb4a_defb4b | -6.2516 | 656 | -- | -- | -- | -- |
| spon1 | -1.8907 | 408 | -- | -- | -- | -- |
| tnfrsf4 | -2.0063 | 308 | -- | -- | -- | -- |
| mzb1 | -2.1165 | 382 | -- | -- | -- | -- |
| dsc2 | -1.6015 | 615 | -- | -- | -- | -- |
| asgr1 | -1.3829 | 576 | -- | -- | -- | -- |
| il10rb | -1.5362 | 366 | -- | -- | -- | -- |
| klk10 | -3.165 | 463 | -- | -- | -- | -- |
| cd4 | -1.1964 | 483 | -- | -- | -- | -- |
| tnfsf13 | -1.5839 | 366 | -- | -- | -- | -- |
| icam1 | -2.3222 | 499 | -- | -- | -- | -- |
| tnfrsf12a | -2.3869 | 398 | -- | -- | -- | -- |
| ambp | -1.8247 | 352 | -- | -- | -- | -- |
| npdc1 | -1.2994 | 455 | -- | -- | -- | -- |
| enpp5 | -4.669 | 366 | -- | -- | -- | -- |
| shisa5 | -1.0913 | 2646 | -- | -- | -- | -- |
| nectin4 | -3.1434 | 398 | -- | -- | -- | -- |
| ccl22 | -2.808 | 562 | -- | -- | -- | -- |
| fabp2 | -3.0391 | 455 | -- | -- | -- | -- |
| fgf5 | -1.8015 | 747 | -- | -- | -- | -- |
| capg | -4.7213 | 568 | -- | -- | -- | -- |
| lrrn1 | -1.9497 | 747 | -- | -- | -- | -- |
| chchd10 | -1.951 | 2676 | -- | -- | -- | -- |
| fas | -1.2636 | 303 | -- | -- | -- | -- |
| cd300e | -1.6122 | 375 | -- | -- | -- | -- |
| angpt2 | -1.6001 | 330 | -- | -- | -- | -- |
| spon2 | -2.0306 | 541 | -- | -- | -- | -- |
| ca12 | -1.5114 | 520 | -- | -- | -- | -- |
| serping1 | -1.2304 | 2654 | -- | -- | -- | -- |
| hspg2 | -1.6113 | 526 | -- | -- | -- | -- |
| tnfrsf21 | -1.5363 | 324 | -- | -- | -- | -- |
| hla_e | -1.5332 | 459 | -- | -- | -- | -- |
| timd4 | -2.2798 | 363 | -- | -- | -- | -- |
| ccl18 | -3.1388 | 659 | -- | -- | -- | -- |
| ceacam6 | -1.3644 | 2646 | -- | -- | -- | -- |
| sel1l | -0.805 | 2983 | -- | -- | -- | -- |
| fap | -1.5587 | 253 | -- | -- | -- | -- |
| dpp6 | -1.7368 | 520 | -- | -- | -- | -- |
| ceacam5 | -3.5062 | 1072 | -- | -- | -- | -- |
| bpifb1 | -3.7648 | 490 | -- | -- | -- | -- |
| oscar | -2.2842 | 408 | -- | -- | -- | -- |
| nbl1 | -3.1971 | 398 | -- | -- | -- | -- |
| cstb | -1.9846 | 695 | -- | -- | -- | -- |
| efemp1 | -1.6436 | 385 | -- | -- | -- | -- |
| rnf149 | -1.5874 | 2983 | -- | -- | -- | -- |
| ifi30 | -2.0262 | 3008 | -- | -- | -- | -- |
| chi3l1 | -2.577 | 770 | -- | -- | -- | -- |
| fam3c | -2.1806 | 490 | -- | -- | -- | -- |
| ptgds | -1.6539 | 550 | -- | -- | -- | -- |
| olr1 | -3.1748 | 602 | -- | -- | -- | -- |
| clec4d | -3.6362 | 459 | -- | -- | -- | -- |
| b4gat1 | -1.3506 | 465 | -- | -- | -- | -- |
| dll1 | -3.1281 | 398 | -- | -- | -- | -- |
| cd300lf | -7.0078 | 330 | -- | -- | -- | -- |
| hyou1 | -1.4719 | 253 | -- | -- | -- | -- |
| timp1 | -2.5825 | 589 | -- | -- | -- | -- |
| tmed4 | -2.1827 | 3096 | -- | -- | -- | -- |
| b2m | -1.5106 | 2621 | -- | -- | -- | -- |
| qpct | -2.5869 | 443 | -- | -- | -- | -- |
| ca4 | -1.5733 | 253 | -- | -- | -- | -- |
| guca2a | -2.5606 | 534 | -- | -- | -- | -- |
| tnfrsf9 | -1.9135 | 576 | -- | -- | -- | -- |
| prss2 | -3.995 | 363 | -- | -- | -- | -- |
| pglyrp1 | -3.5522 | 328 | -- | -- | -- | -- |
| btn2a1 | -5.088 | 366 | -- | -- | -- | -- |
| cd79b | -1.9584 | 287 | -- | -- | -- | -- |
| vmo1 | -5.3471 | 520 | -- | -- | -- | -- |
| igdcc4 | -1.6821 | 2646 | -- | -- | -- | -- |

Fill value: minima of columns; Fill the quantity: number of participants with missing protein expression values; The validation set only interpolated the proteins screened by LASSO.

# Supplementary table 5. Coefficients of candidate proteins for constructing protein risk scores in men and women.

| **Protein** | **Coefficient (Male)** | **Protein** | **Coefficient (Female)** |
| --- | --- | --- | --- |
| mmp12 | 0.678296 | tnfrsf10b | 0.426176 |
| cxcl17 | 0.221177 | mmp12 | 0.390088 |
| il6 | 0.06232 | ntprobnp | 0.185905 |
| igfbpl1 | 0.015989 | plaur | 0.157299 |
| cfc1 | 0.015062 | alpp | 0.123086 |
| ntprobnp | 0.011665 | msln | 0.078726 |
| adgrg1 | 0.065963 | cxcl17 | 0.072846 |
| s100a12 | 0.006549 | cst7 | 0.060735 |
| tnfrsf11b | 0.005604 | il6 | 0.032959 |
| vstm2l | 0.004269 | gal | -0.00645 |
| mln | 0.000108 | ret | -0.04222 |
| ca14 | -0.05763 | cbln4 | -0.19342 |
| ctsv | -0.17255 | ncan | -0.67849 |
| egfr | -0.23189 | -- | -- |

# Supplementary table 6. Proteins Selected by LASSO Cox Regression and Their Corresponding Coefficients in the Sensitivity Analysis Using K-Nearest Neighbors (KNN) Imputation, Stratified by Sex.

| **Protein** | **Coefficient (Male)** | **Protein** | **Coefficient (Female)** |
| --- | --- | --- | --- |
| mmp12 | 0.779927 | tnfrsf10b | 0.420425 |
| cxcl17 | 0.176744 | mmp12 | 0.370169 |
| il6 | 0.004918 | ntprobnp | 0.171893 |
| s100a12 | 0.054673 | plaur | 0.169054 |
| igdcc4 | -0.14157 | alpp | 0.115938 |
| tnfrsf11b | 0.155738 | msln | 0.07986 |
| mln | 0.015405 | cxcl17 | 0.064739 |
| adgrg1 | 0.051932 | cst7 | 0.039943 |
| fgfbp1 | -0.14001 | il6 | 0.008422 |
| prune2 | 0.12258 | cbln4 | -0.12461 |
| st8sia1 | 0.058863 | ncan | -0.64689 |
| bpifb1 | 0.01717 |  |  |
| ctsv | -0.17447 |  |  |
| nppb | 0.014267 |  |  |
| lpa | 0.047229 |  |  |
| cdan1 | 0.001897 |  |  |
| ca14 | -0.03305 |  |  |
| smad3 | -0.11975 |  |  |
| notch2 | -0.04663 |  |  |
| vstm2l | 0.05351 |  |  |
| boc | -0.17662 |  |  |
| slc4a1 | -0.08702 |  |  |

# Supplementary table 7. Sensitivity analysis using Fine-Gray subdistribution hazard models to assess the associations between traditional clinical risk factors and incident AA.

|  | **Male** |  | **Female** |  |
| --- | --- | --- | --- | --- |
|  | **HR (95% CI)** | **P-value** | **HR (95% CI)** | **P-value** |
| Low SES | 1.06 (0.98-1.14) | 0.120 | 1.09 (0.94-1.26) | 0.250 |
| Obesity | 1.26 (1.16-1.36) | <0.001 | 1.03 (0.88-1.21) | 0.240 |
| Current smoker | 2.79 (2.58-3.04) | <0.001 | 4.36 (3.69-5.15) | <0.001 |
| Hypertension | 1.29 (1.15-1.44) | <0.001 | 1.30 (1.02-1.65) | <0.001 |
| Hyperlipidemia | 1.11 (1.00-1.24) | 0.048 | 1.20 (0.98-1.56) | 0.160 |
| Coronary heart disease | 1.52 (1.37-1.68) | <0.001 | 1.78 (1.21-2.45) | <0.001 |
| Stroke | 1.21 (1.02-1.44) | 0.037 | 1.72 (1.21-2.45) | 0.026 |
| Valvular heart disease | 2.08 (1.67-2.59) | <0.001 | 4.57 (3.19-6.54) | <0.001 |

Model 1 was adjusted for age, SES, smoking status, obesity, hypertension, hyperlipidemia, coronary heart disease, stroke, and valvular heart disease. Model 2 was further adjusted for the use of antihypertensive and lipid-lowering medications. The Fine-Gray competing risk model was adjusted for the same covariates as Model 2, with all-cause mortality specified as the competing event. HR, hazard ratio; CI, confidence interval.

# Supplementary table 8. Associations between clinical risk factors and incident AA, AAA, and TAA in male participants.

|  | **AA** | | | | **AAA** | | | | **TAA** | | | |
| --- | --- | --- | --- | --- | --- | --- | --- | --- | --- | --- | --- | --- |
|  | **Model 1** |  | **Model 2** |  | **Model 1** |  | **Model 2** |  | **Model 1** |  | **Model 2** |  |
|  | **HR (95% CI)** | **P-value** | **HR (95% CI)** | **P-value** | **HR (95% CI)** | **P-value** | **HR (95% CI)** | **P-value** | **HR (95% CI)** | **P-value** | **HR (95% CI)** | **P-value** |
| Low SES | 1.07 (0.99-1.15) | 0.086 | 1.07 (0.99-1.15) | 0.084 | 1.12 (0.97-1.23) | 0.084 | 1.12 (0.97-1.24) | 0.088 | 1.00 (0.84-1.15) | 0.826 | 0.99 (0.84-1.15) | 0.863 |
| Obesity | 1.27 (1.18-1.38) | <0.001 | 1.27 (1.17-1.37) | <0.001 | 1.37 (1.24-1.51) | <0.001 | 1.37 (1.24-1.51) | <0.001 | 1.16 (0.97-1.38) | 0.097 | 1.15 (0.97-1.37) | 0.125 |
| Current smoker | 2.81 (2.58-3.06) | <0.001 | 2.82 (2.59-3.07) | <0.001 | 4.42 (4.00-4.88) | <0.001 | 4.42 (4.00-4.88) | <0.001 | 0.96 (0.74-1.25) | 0.775 | 0.96 (0.74-1.26) | 0.839 |
| Hypertension | 1.42 (1.31-1.54) | <0.001 | 1.29 (1.15-1.44) | <0.001 | 1.38 (1.25-1.53) | <0.001 | 1.30 (1.14-1.49) | <0.001 | 1.62 (1.36-1.92) | <0.001 | 1.28 (1.01-1.64) | 0.044 |
| Hyperlipidemia | 1.19 (1.09-1.29) | <0.001 | 1.11 (1.00-1.23) | 0.047 | 1.33 (1.20-1.47) | <0.001 | 1.19 (1.05-1.35) | 0.008 | 0.79 (0.64-1.12) | 0.054 | 0.83 (0.64-1.08) | 0.160 |
| Coronary heart disease | 1.57 (1.43-1.74) | <0.001 | 1.50 (1.36-1.67) | <0.001 | 2.02 (1.81-2.27) | <0.001 | 1.91 (1.69-2.15) | <0.001 | 0.75 (0.56-1.00) | 0.051 | 0.72 (0.54-1.04) | 0.084 |
| Stroke | 1.23 (1.04-1.46) | 0.018 | 1.21 (1.02-1.44) | 0.031 | 1.53 (1.26-1.87) | <0.001 | 1.50 (1.23-1.83) | <0.001 | 1.01 (0.63-1.61) | 0.965 | 1.01 (0.63-1.62) | 0.975 |
| Valvular heart disease | 2.00 (1.61-2.49) | <0.001 | 2.01 (1.61-2.49) | <0.001 | 0.91 (0.63-1.32) | 0.624 | 0.91 (0.63-1.33) | 0.637 | 10.5 (7.73-14.10) | <0.001 | 8.91 (6.47-12.30) | <0.001 |

Model 1 was adjusted for age, SES, smoking status, obesity, hypertension, hyperlipidemia, coronary heart disease, stroke, and valvular heart disease. Model 2 was further adjusted for the use of antihypertensive and lipid-lowering medications. HR, hazard ratio; CI, confidence interval.

# Supplementary table 9. Associations between clinical risk factors and incident AA, AAA, and TAA in female participants.

|  | **AA** | | | | **AAA** | | | | **TAA** | | | |
| --- | --- | --- | --- | --- | --- | --- | --- | --- | --- | --- | --- | --- |
|  | **Model 1** |  | **Model 2** |  | **Model 1** |  | **Model 2** |  | **Model 1** |  | **Model 2** |  |
|  | **HR (95% CI)** | **P-value** | **HR (95% CI)** | **P-value** | **HR (95% CI)** | **P-value** | **HR (95% CI)** | **P-value** | **HR (95% CI)** | **P-value** | **HR (95% CI)** | **P-value** |
| Low SES | 1.09 (0.94-1.26) | 0.233 | 1.09 (0.95-1.26) | 0.229 | 1.25 (1.00-1.56) | 0.053 | 1.24 (0.99-1.56) | 0.057 | 1.05 (0.83-1.33) | 0.673 | 1.06 (0.84-1.33) | 0.649 |
| Obesity | 1.04 (0.89-1.22) | 0.632 | 1.03 (0.88-1.21) | 0.697 | 1.07 (0.84-1.36) | 0.589 | 1.05 (0.83-1.34) | 0.672 | 0.97 (0.74-1.27) | 0.833 | 0.96 (0.73-1.27) | 0.789 |
| Current smoker | 4.38 (3.72-5.16) | <0.001 | 4.42 (3.76-5.21) | <0.001 | 9.57 (7.67-11.90) | <0.001 | 9.54 (7.65-11.90) | <0.001 | 2.10 (1.49-2.96) | <0.001 | 2.14 (1.52-3.02) | <0.001 |
| Hypertension | 1.63 (1.40-1.91) | <0.001 | 1.30 (1.03-1.64) | 0.026 | 1.47 (1.16-1.86) | <0.001 | 1.23 (0.88-1.74) | 0.229 | 1.77 (1.38-2.28) | 0.213 | 1.28 (0.87-1.89) | 0.213 |
| Hyperlipidemia | 1.16 (0.97-1.38) | 0.112 | 1.20 (0.95-1.52) | 0.129 | 1.48 (1.14-1.90) | <0.001 | 1.11 (0.80-1.55) | 0.528 | 0.66 (0.47-0.93) | 0.017 | 0.92 (0.59-1.43) | 0.695 |
| Coronary heart disease | 1.79 (1.42-2.26) | <0.001 | 1.78 (1.41-2.26) | <0.001 | 2.60 (1.92-3.53) | <0.001 | 2.37 (1.74-3.23) | <0.001 | 1.44 (0.91-2.26) | 0.116 | 1.56 (0.98-2.48) | 0.058 |
| Stroke | 1.73 (1.23-2.44) | 0.002 | 1.73 (1.23-2.44) | 0.002 | 2.12 (1.35-3.34) | 0.001 | 2.01 (1.27-3.16) | 0.003 | 1.75 (0.92-3.34) | 0.089 | 1.95 (0.99–3.85) | 0.095 |
| Valvular heart disease | 4.59 (3.26-6.46) | <0.001 | 4.62 (3.29-6.50) | <0.001 | 1.90 (0.96-3.75) | 0.065 | 1.95 (1.04-3.85) | 0.045 | 9.82 (6.16-15.70) | <0.001 | 9.77 (6.12-15.60) | <0.001 |

Model 1 was adjusted for age, SES, smoking status, obesity, hypertension, hyperlipidemia, coronary heart disease, stroke, and valvular heart disease. Model 2 was further adjusted for the use of antihypertensive and lipid-lowering medications. HR, hazard ratio; CI, confidence interval.

# Supplementary table 10. 5-year,10-year and 15-year PAR of Risk Factors in Men.

|  | **Population attributable risk (PAR)** | | | | | | | | | | | |
| --- | --- | --- | --- | --- | --- | --- | --- | --- | --- | --- | --- | --- |
|  | **5-year** | | | | **10-year** | | | | **15-year** | | | |
| **Risk Factors** | **PAR** | **95%LCI** | **95%UCI** | **P-Value** | **PAR** | **95%LCI** | **95%UCI** | **P-Value** | **PAR** | **95%LCI** | **95%UCI** | **P-Value** |
| Low SES | 3.40% | -0.46% | 7.26% | 0.084 | 3.38% | -0.45% | 7.21% | 0.084 | 3.34% | -0.45% | 7.14% | 0.084 |
| Obesity | 6.77% | 4.32% | 9.21% | <0.001 | 6.72% | 4.29% | 9.14% | <0.001 | 6.65% | 4.25% | 9.05% | <0.001 |
| Current smoke | 15.62% | 13.88% | 17.37% | <0.001 | 15.49% | 13.77% | 17.22% | <0.001 | 15.30% | 13.60% | 16.99% | <0.001 |
| Hypertension | 11.43% | 6.70% | 16.16% | <0.001 | 11.34% | 6.64% | 16.03% | <0.001 | 11.20% | 6.55% | 15.85% | <0.001 |
| Hyperlipidemia | 3.52% | 0.09% | 6.95% | 0.044 | 3.48% | 0.09% | 6.88% | 0.044 | 3.43% | 0.08% | 6.77% | 0.045 |
| Coronary heart disease | 7.16% | 5.24% | 9.08% | <0.001 | 7.07% | 5.18% | 8.97% | <0.001 | 6.94% | 5.09% | 8.80% | <0.001 |
| Stroke | 0.81% | -0.01% | 1.63% | 0.053 | 0.80% | -0.01% | 1.61% | 0.052 | 0.78% | -0.01% | 1.57% | 0.052 |
| Valvular heart disease | 1.59% | 0.90% | 2.28% | <0.001 | 1.56% | 0.88% | 2.23% | 0.000 | 1.51% | 0.86% | 2.15% | <0.001 |

Low SES: High Townsend deprivation index (TDI); Obesity: BMI >=30.0kg/m2; AA: Aortic aneurysm. CI, confidence interval.

# Supplementary table 11. 5-year,10-year and 15-year PAR of Risk Factors in Women.

|  | **Population attributable risk (PAR)** | | | | | | | | | | | |
| --- | --- | --- | --- | --- | --- | --- | --- | --- | --- | --- | --- | --- |
|  | **5-year** | | | | **10-year** | | | | **15-year** | | | |
| **Risk Factors** | **PAR** | **95%LCI** | **95%UCI** | **P-Value** | **PAR** | **95%LCI** | **95%UCI** | **P-Value** | **PAR** | **95%LCI** | **95%UCI** | **P-Value** |
| Low SES | 4.71% | -2.92% | 12.34% | 0.227 | 4.69% | -2.92% | 12.31% | 0.227 |  |  |  |  |
| Obesity | 0.83% | -3.62% | 5.27% | 0.715 | 0.83% | -3.63% | 5.29% | 0.715 | 0.82% | -3.60% | 5.25% | 0.715 |
| Current smoke | 20.09% | 16.74% | 23.44% | <0.001 | 20.01% | 16.68% | 23.34% | <0.001 | 19.88% | 16.58% | 23.18% | <0.001 |
| Hypertension | 10.27% | 1.65% | 18.88% | 0.019 | 10.23% | 1.64% | 18.82% | 0.020 | 10.17% | 1.62% | 18.71% | 0.020 |
| Hyperlipidemia | 4.45% | -1.55% | 10.44% | 0.146 | 4.42% | -1.54% | 10.39% | 0.146 | 4.38% | -1.53% | 10.29% | 0.146 |
| Coronary heart disease | 5.59% | 2.85% | 8.34% | <0.001 | 5.54% | 2.82% | 8.26% | <0.001 | 5.45% | 2.78% | 8.13% | <0.001 |
| Stroke | 1.89% | 0.35% | 3.43% | 0.016 | 1.87% | 0.35% | 3.40% | 0.016 | 1.84% | 0.35% | 3.33% | 0.016 |
| Valvular heart disease | 3.77% | 2.10% | 5.43% | <0.001 | 3.71% | 2.09% | 5.34% | <0.001 | 3.62% | 2.06% | 5.18% | <0.001 |

Low SES: High Townsend deprivation index (TDI); Obesity: BMI >=30.0kg/m2; AA: Aortic aneurysm. CI, confidence interval.

# Supplementary table 12. Aassociation between proteins and AA in men.

| **Protein** | **HR** | **95%LCI** | **95%UCI** | **P-Value** | **pBH** |
| --- | --- | --- | --- | --- | --- |
| mmp12 | 2.706903 | 2.393523 | 3.061313 | 1.15E-56 | 3.35E-53 |
| cxcl17 | 2.018114 | 1.774302 | 2.295429 | 1.15E-26 | 1.68E-23 |
| wfdc2 | 1.972266 | 1.686747 | 2.306116 | 1.70E-17 | 1.66E-14 |
| npc2 | 2.208839 | 1.81151 | 2.693317 | 4.79E-15 | 3.50E-12 |
| plaur | 2.675118 | 2.052399 | 3.486776 | 3.38E-13 | 1.98E-10 |
| il6 | 1.357074 | 1.248783 | 1.474755 | 6.20E-13 | 3.02E-10 |
| prss8 | 2.020004 | 1.666942 | 2.447845 | 7.32E-13 | 3.06E-10 |
| ca6 | 0.59486 | 0.515673 | 0.686206 | 1.03E-12 | 3.76E-10 |
| hgf | 2.00823 | 1.647055 | 2.448606 | 5.47E-12 | 1.78E-09 |
| ccl7 | 1.505613 | 1.338409 | 1.693706 | 9.56E-12 | 2.62E-09 |
| cst3 | 2.404381 | 1.867781 | 3.095142 | 9.85E-12 | 2.62E-09 |
| lamp3 | 1.704286 | 1.459156 | 1.990596 | 1.71E-11 | 4.16E-09 |
| msln | 1.550508 | 1.359139 | 1.768822 | 6.78E-11 | 1.52E-08 |
| alpp | 1.249298 | 1.167024 | 1.337373 | 1.52E-10 | 3.17E-08 |
| spp1 | 1.806212 | 1.504475 | 2.168464 | 2.30E-10 | 4.49E-08 |
| orm1 | 4.339811 | 2.750198 | 6.848218 | 2.85E-10 | 4.90E-08 |
| tnfrsf10b | 1.396302 | 1.258706 | 1.548939 | 2.85E-10 | 4.90E-08 |
| col18a1 | 3.086866 | 2.170619 | 4.389874 | 3.53E-10 | 5.73E-08 |
| adm | 2.763937 | 2.010649 | 3.799442 | 3.80E-10 | 5.84E-08 |
| ifi30 | 2.059569 | 1.641149 | 2.584668 | 4.51E-10 | 6.58E-08 |
| cd83 | 2.051493 | 1.636202 | 2.572191 | 4.77E-10 | 6.64E-08 |
| igfbp4 | 1.614265 | 1.382328 | 1.885118 | 1.44E-09 | 1.91E-07 |
| rnase1 | 2.351131 | 1.773372 | 3.117122 | 2.83E-09 | 3.44E-07 |
| rnase6 | 1.797941 | 1.481914 | 2.181362 | 2.71E-09 | 3.44E-07 |
| pgf | 2.30804 | 1.748687 | 3.046312 | 3.49E-09 | 4.08E-07 |
| gdf15 | 1.554619 | 1.340726 | 1.802636 | 5.14E-09 | 5.78E-07 |
| ccl16 | 1.747729 | 1.446928 | 2.111064 | 6.89E-09 | 7.45E-07 |
| trem2 | 1.583586 | 1.354106 | 1.851957 | 8.65E-09 | 9.03E-07 |
| shisa5 | 1.945424 | 1.540089 | 2.457438 | 2.37E-08 | 2.39E-06 |
| ggh | 1.923655 | 1.527906 | 2.421908 | 2.59E-08 | 2.52E-06 |
| prap1 | 2.119158 | 1.626082 | 2.761749 | 2.73E-08 | 2.58E-06 |
| tnfrsf9 | 1.523437 | 1.313059 | 1.767522 | 2.82E-08 | 2.58E-06 |
| ceacam5 | 1.416482 | 1.251198 | 1.6036 | 3.80E-08 | 3.36E-06 |
| acvrl1 | 2.22615 | 1.672188 | 2.96363 | 4.22E-08 | 3.52E-06 |
| ocln | 1.692165 | 1.40212 | 2.042209 | 4.18E-08 | 3.52E-06 |
| asgr1 | 2.000166 | 1.55693 | 2.569584 | 5.84E-08 | 4.74E-06 |
| rarres2 | 1.516427 | 1.302558 | 1.76541 | 7.97E-08 | 6.13E-06 |
| s100a12 | 1.396084 | 1.235946 | 1.576971 | 7.97E-08 | 6.13E-06 |
| edn1 | 2.396547 | 1.740195 | 3.300456 | 8.66E-08 | 6.49E-06 |
| vwc2 | 1.80973 | 1.4549 | 2.251099 | 9.97E-08 | 7.29E-06 |
| f9 | 3.537364 | 2.208275 | 5.66639 | 1.48E-07 | 1.05E-05 |
| il2ra | 1.650219 | 1.368441 | 1.99002 | 1.58E-07 | 1.10E-05 |
| scarb2 | 1.791415 | 1.439348 | 2.229598 | 1.77E-07 | 1.20E-05 |
| rnase4 | 2.178201 | 1.625781 | 2.918327 | 1.83E-07 | 1.21E-05 |
| ctsl | 2.495747 | 1.766734 | 3.525576 | 2.11E-07 | 1.32E-05 |
| efna4 | 1.802463 | 1.442904 | 2.251621 | 2.10E-07 | 1.32E-05 |
| il7r | 0.641946 | 0.543119 | 0.758757 | 2.03E-07 | 1.32E-05 |
| xcl1 | 1.442399 | 1.25494 | 1.65786 | 2.51E-07 | 1.53E-05 |
| b2m | 1.583496 | 1.328251 | 1.887791 | 2.97E-07 | 1.77E-05 |
| vsig4 | 1.645246 | 1.359795 | 1.99062 | 3.04E-07 | 1.78E-05 |
| vsig2 | 1.42349 | 1.242927 | 1.630284 | 3.36E-07 | 1.92E-05 |
| lilrb4 | 1.635933 | 1.352529 | 1.978719 | 3.95E-07 | 2.22E-05 |
| tnfrsf10a | 1.681428 | 1.373468 | 2.05844 | 4.79E-07 | 2.64E-05 |
| lgals9 | 1.953424 | 1.503582 | 2.537849 | 5.33E-07 | 2.88E-05 |
| igfbp7 | 1.743692 | 1.402011 | 2.168642 | 5.83E-07 | 3.07E-05 |
| sftpa2 | 1.476141 | 1.266904 | 1.719936 | 5.93E-07 | 3.07E-05 |
| tnfrsf1b | 1.523032 | 1.291113 | 1.796609 | 6.00E-07 | 3.07E-05 |
| st6gal1 | 1.786899 | 1.422225 | 2.24508 | 6.22E-07 | 3.13E-05 |
| egfr | 0.240612 | 0.137035 | 0.422479 | 7.06E-07 | 3.50E-05 |
| tnfrsf1a | 1.830424 | 1.440548 | 2.325817 | 7.54E-07 | 3.67E-05 |
| havcr2 | 1.769804 | 1.40986 | 2.221643 | 8.62E-07 | 4.13E-05 |
| tff2 | 1.406671 | 1.227748 | 1.611669 | 8.83E-07 | 4.16E-05 |
| lair1 | 1.589844 | 1.318589 | 1.9169 | 1.19E-06 | 5.52E-05 |
| colec12 | 2.021085 | 1.519243 | 2.688698 | 1.35E-06 | 6.18E-05 |
| ceacam6 | 1.49179 | 1.26726 | 1.7561 | 1.54E-06 | 6.62E-05 |
| hla_e | 1.932655 | 1.477608 | 2.527838 | 1.51E-06 | 6.62E-05 |
| msr1 | 1.587445 | 1.315142 | 1.916128 | 1.49E-06 | 6.62E-05 |
| plat | 1.513627 | 1.278216 | 1.792395 | 1.54E-06 | 6.62E-05 |
| tff3 | 1.506524 | 1.274048 | 1.781421 | 1.65E-06 | 6.99E-05 |
| col6a3 | 1.646554 | 1.338939 | 2.024842 | 2.29E-06 | 9.55E-05 |
| igdcc4 | 0.446077 | 0.31886 | 0.624051 | 2.45E-06 | 1.00E-04 |
| smoc1 | 1.829957 | 1.423183 | 2.352995 | 2.46E-06 | 1.00E-04 |
| tnfrsf11b | 1.985546 | 1.491722 | 2.642848 | 2.59E-06 | 0.000104 |
| cxcl16 | 2.397553 | 1.664056 | 3.454367 | 2.69E-06 | 0.000106 |
| tnfrsf6b | 1.393515 | 1.212919 | 1.601002 | 2.79E-06 | 0.000109 |
| clec5a | 1.942165 | 1.47077 | 2.564647 | 2.87E-06 | 0.000111 |
| cd80 | 1.878317 | 1.441652 | 2.447244 | 3.02E-06 | 0.000115 |
| fam20a | 2.080514 | 1.529018 | 2.830928 | 3.13E-06 | 0.000117 |
| cfi | 3.502805 | 2.06439 | 5.943473 | 3.37E-06 | 0.000125 |
| mmp7 | 1.55684 | 1.289931 | 1.878977 | 3.97E-06 | 0.000145 |
| crip2 | 1.545337 | 1.284048 | 1.859795 | 4.11E-06 | 0.000148 |
| rnf149 | 2.037046 | 1.504338 | 2.758394 | 4.22E-06 | 0.000151 |
| cd74 | 1.736437 | 1.36855 | 2.203218 | 5.55E-06 | 0.000195 |
| dpp6 | 0.536964 | 0.41046 | 0.702456 | 5.72E-06 | 0.000199 |
| enpp5 | 0.690958 | 0.588863 | 0.810754 | 5.85E-06 | 0.000201 |
| spink1 | 1.539844 | 1.277068 | 1.85669 | 6.13E-06 | 0.000208 |
| rnaset2 | 2.027349 | 1.490627 | 2.757325 | 6.66E-06 | 0.000224 |
| pigr | 1.658666 | 1.330484 | 2.0678 | 6.85E-06 | 0.000227 |
| angpt2 | 1.622789 | 1.313949 | 2.004221 | 6.96E-06 | 0.000229 |
| tnf | 1.497478 | 1.254833 | 1.787042 | 7.58E-06 | 0.000246 |
| cd27 | 1.532514 | 1.270911 | 1.847965 | 7.81E-06 | 0.000248 |
| klk4 | 1.328132 | 1.172824 | 1.504006 | 7.73E-06 | 0.000248 |
| sel1l | 1.587774 | 1.294978 | 1.946772 | 8.77E-06 | 0.000276 |
| inhbb | 1.518177 | 1.262755 | 1.825264 | 8.91E-06 | 0.000277 |
| eda2r | 1.571828 | 1.287116 | 1.919519 | 9.19E-06 | 0.000283 |
| gpr37 | 1.365645 | 1.18978 | 1.567504 | 9.40E-06 | 0.000286 |
| mln | 1.271786 | 1.143075 | 1.41499 | 1.00E-05 | 0.000303 |
| tnfrsf13b | 1.509485 | 1.257223 | 1.812363 | 1.02E-05 | 0.000303 |
| reg1b | 1.371703 | 1.191937 | 1.578581 | 1.03E-05 | 0.000306 |
| igfbpl1 | 1.708716 | 1.34569 | 2.169674 | 1.10E-05 | 0.000322 |
| clec4d | 1.36948 | 1.190036 | 1.575984 | 1.14E-05 | 0.000331 |
| ckap4 | 1.762304 | 1.367537 | 2.271028 | 1.19E-05 | 0.000335 |
| ntprobnp | 1.172496 | 1.091931 | 1.259004 | 1.18E-05 | 0.000335 |
| ptgds | 1.740686 | 1.358276 | 2.230758 | 1.19E-05 | 0.000335 |
| cfb | 2.060506 | 1.490551 | 2.848401 | 1.21E-05 | 0.000337 |
| adgrg1 | 1.201157 | 1.106348 | 1.304091 | 1.25E-05 | 0.000344 |
| psap | 1.881084 | 1.416492 | 2.498056 | 1.27E-05 | 0.000346 |
| furin | 1.802614 | 1.38042 | 2.353932 | 1.51E-05 | 0.000408 |
| relt | 1.761111 | 1.361621 | 2.277809 | 1.62E-05 | 0.000435 |
| cd300lf | 1.433779 | 1.217067 | 1.68908 | 1.64E-05 | 0.000435 |
| bsg | 2.454415 | 1.630412 | 3.694866 | 1.69E-05 | 0.000441 |
| fgf23 | 1.334417 | 1.170005 | 1.521933 | 1.71E-05 | 0.000441 |
| gm2a | 1.529063 | 1.260053 | 1.855505 | 1.70E-05 | 0.000441 |
| gchfr | 1.461974 | 1.229241 | 1.738771 | 1.76E-05 | 0.000452 |
| cpxm2 | 2.205015 | 1.534946 | 3.167599 | 1.88E-05 | 0.000478 |
| havcr1 | 1.299452 | 1.152206 | 1.465517 | 1.96E-05 | 0.000495 |
| cdcp1 | 1.380317 | 1.190197 | 1.600806 | 2.02E-05 | 0.000504 |
| il10rb | 1.791685 | 1.369824 | 2.343465 | 2.07E-05 | 0.000508 |
| serpina1 | 5.385234 | 2.480704 | 11.69053 | 2.07E-05 | 0.000508 |
| cfh | 2.671804 | 1.695008 | 4.211506 | 2.31E-05 | 0.000562 |
| lrg1 | 2.227664 | 1.536617 | 3.229488 | 2.37E-05 | 0.000567 |
| siglec7 | 1.922125 | 1.419801 | 2.602171 | 2.36E-05 | 0.000567 |
| sit1 | 1.406403 | 1.20056 | 1.647538 | 2.40E-05 | 0.000571 |
| ang | 1.72181 | 1.337072 | 2.217256 | 2.54E-05 | 0.000596 |
| csf1 | 1.949556 | 1.42878 | 2.660148 | 2.55E-05 | 0.000596 |
| fgfbp1 | 0.56737 | 0.435571 | 0.73905 | 2.65E-05 | 0.000614 |
| gcnt1 | 1.72903 | 1.338753 | 2.233083 | 2.73E-05 | 0.000628 |
| ctsd | 1.490834 | 1.236629 | 1.797294 | 2.83E-05 | 0.000643 |
| spink2 | 1.748793 | 1.346122 | 2.271918 | 2.84E-05 | 0.000643 |
| cfd | 2.367582 | 1.58083 | 3.545887 | 2.89E-05 | 0.000646 |
| guk1 | 1.491423 | 1.23661 | 1.798742 | 2.89E-05 | 0.000646 |
| gfra1 | 1.81618 | 1.372875 | 2.402629 | 2.92E-05 | 0.000647 |
| sorcs2 | 1.681469 | 1.316387 | 2.147801 | 3.17E-05 | 0.000696 |
| lrrn1 | 0.616774 | 0.491078 | 0.774642 | 3.24E-05 | 0.000706 |
| il18bp | 1.745549 | 1.341901 | 2.270617 | 3.30E-05 | 0.000714 |
| icam1 | 1.841605 | 1.378744 | 2.459856 | 3.56E-05 | 0.000764 |
| pon3 | 0.657015 | 0.538165 | 0.802113 | 3.69E-05 | 0.000788 |
| ccl22 | 1.404615 | 1.194729 | 1.651373 | 3.88E-05 | 0.000821 |
| agrn | 1.575471 | 1.267663 | 1.95802 | 4.16E-05 | 0.000875 |
| chchd10 | 1.52191 | 1.244832 | 1.860662 | 4.21E-05 | 0.000878 |
| cd59 | 1.961735 | 1.418388 | 2.713226 | 4.66E-05 | 0.000958 |
| prss2 | 1.338241 | 1.163167 | 1.539667 | 4.64E-05 | 0.000958 |
| il1rn | 1.334396 | 1.161231 | 1.533384 | 4.75E-05 | 0.000964 |
| serpinf1 | 2.146602 | 1.4857 | 3.101503 | 4.73E-05 | 0.000964 |
| apom | 0.537166 | 0.398005 | 0.724985 | 4.86E-05 | 0.00098 |
| prune2 | 1.781126 | 1.347218 | 2.354786 | 5.07E-05 | 0.001016 |
| itih3 | 1.657077 | 1.296425 | 2.118059 | 5.51E-05 | 0.001095 |
| edil3 | 1.591043 | 1.269327 | 1.9943 | 5.60E-05 | 0.001106 |
| timp1 | 1.744987 | 1.33031 | 2.288925 | 5.78E-05 | 0.001134 |
| fstl3 | 1.631439 | 1.284605 | 2.071917 | 5.98E-05 | 0.001165 |
| reg1a | 1.390559 | 1.183049 | 1.634466 | 6.37E-05 | 0.001234 |
| pga4 | 1.317382 | 1.150066 | 1.509041 | 6.96E-05 | 0.001339 |
| il22 | 1.311347 | 1.147283 | 1.498872 | 7.05E-05 | 0.001346 |
| septin3 | 1.278412 | 1.132514 | 1.443106 | 7.11E-05 | 0.001349 |
| cdh2 | 1.650797 | 1.286691 | 2.117937 | 8.06E-05 | 0.00152 |
| lepr | 0.550674 | 0.40907 | 0.741294 | 8.36E-05 | 0.001567 |
| rspo1 | 1.673027 | 1.292984 | 2.164775 | 9.06E-05 | 0.001687 |
| ulbp2 | 1.537543 | 1.23947 | 1.907297 | 9.13E-05 | 0.00169 |
| siglec1 | 1.497984 | 1.223325 | 1.83431 | 9.21E-05 | 0.001693 |
| capg | 1.338082 | 1.156184 | 1.548598 | 9.36E-05 | 0.001709 |
| rtn4r | 1.586437 | 1.257771 | 2.000985 | 9.77E-05 | 0.001774 |
| pik3ip1 | 1.703543 | 1.302628 | 2.227848 | 9.98E-05 | 0.0018 |
| mzb1 | 1.383457 | 1.173881 | 1.630449 | 0.000108 | 0.001919 |
| reg3a | 1.288438 | 1.13334 | 1.46476 | 0.000108 | 0.001919 |
| creld1 | 1.727024 | 1.309412 | 2.277824 | 0.000109 | 0.001919 |
| dner | 0.500871 | 0.352844 | 0.710999 | 0.00011 | 0.001919 |
| penk | 1.668151 | 1.287277 | 2.161717 | 0.000109 | 0.001919 |
| pzp | 1.705511 | 1.300921 | 2.235929 | 0.000112 | 0.001929 |
| st8sia1 | 1.347896 | 1.158542 | 1.568199 | 0.000111 | 0.001929 |
| tnfsf13b | 1.711078 | 1.302367 | 2.248051 | 0.000115 | 0.001973 |
| fut3_fut5 | 1.452329 | 1.20092 | 1.756371 | 0.000119 | 0.002037 |
| edar | 0.810432 | 0.72805 | 0.902136 | 0.000122 | 0.002066 |
| hspg2 | 1.733499 | 1.308786 | 2.296035 | 0.000125 | 0.002107 |
| siglec8 | 1.562925 | 1.243759 | 1.963994 | 0.000127 | 0.002138 |
| tff1 | 1.220047 | 1.10188 | 1.350886 | 0.00013 | 0.00217 |
| tmprss11d | 1.581745 | 1.25052 | 2.0007 | 0.000131 | 0.002174 |
| fabp3 | 1.388348 | 1.172968 | 1.643276 | 0.000136 | 0.002251 |
| mfap5 | 1.525444 | 1.227682 | 1.895427 | 0.000138 | 0.00227 |
| eln | 1.762958 | 1.316498 | 2.360824 | 0.000141 | 0.002311 |
| ambp | 2.270406 | 1.487395 | 3.46562 | 0.000145 | 0.002351 |
| lcn2 | 1.51 | 1.22073 | 1.867816 | 0.000146 | 0.002354 |
| sftpd | 1.296458 | 1.133613 | 1.482696 | 0.00015 | 0.002408 |
| chga | 1.217504 | 1.099322 | 1.348391 | 0.000158 | 0.002529 |
| cd70 | 1.465661 | 1.201678 | 1.787634 | 0.000161 | 0.00256 |
| met | 0.406898 | 0.254492 | 0.650576 | 0.000173 | 0.002735 |
| oscar | 1.658991 | 1.27262 | 2.162666 | 0.000182 | 0.002868 |
| ogn | 1.440842 | 1.189731 | 1.744955 | 0.000185 | 0.002899 |
| icam5 | 1.443168 | 1.189706 | 1.750629 | 0.000197 | 0.003064 |
| aldh3a1 | 1.237105 | 1.105638 | 1.384205 | 0.000206 | 0.003182 |
| il18r1 | 1.628739 | 1.25768 | 2.109273 | 0.000217 | 0.003343 |
| lta4h | 1.303193 | 1.132048 | 1.500212 | 0.000227 | 0.003478 |
| ncan | 0.656727 | 0.52463 | 0.822085 | 0.000243 | 0.003696 |
| npl | 1.4984 | 1.207039 | 1.860091 | 0.000247 | 0.003718 |
| pla2g15 | 1.793087 | 1.312234 | 2.450142 | 0.000247 | 0.003718 |
| cd300e | 1.442081 | 1.185133 | 1.754739 | 0.000256 | 0.003833 |
| ccl14 | 1.440821 | 1.184029 | 1.753308 | 0.000266 | 0.003962 |
| fga | 1.877057 | 1.337836 | 2.633612 | 0.000268 | 0.003976 |
| agrp | 1.398403 | 1.167067 | 1.675596 | 0.000279 | 0.004023 |
| cd207 | 1.544855 | 1.222171 | 1.952735 | 0.000274 | 0.004023 |
| cfhr5 | 1.648838 | 1.259027 | 2.159339 | 0.000279 | 0.004023 |
| gfral | 0.706366 | 0.585696 | 0.851897 | 0.000276 | 0.004023 |
| iglc2 | 1.634788 | 1.254187 | 2.130887 | 0.000278 | 0.004023 |
| lmnb2 | 1.779068 | 1.304457 | 2.42636 | 0.000274 | 0.004023 |
| gipc2 | 1.74655 | 1.292273 | 2.36052 | 0.000285 | 0.004089 |
| lgals4 | 1.312618 | 1.133068 | 1.52062 | 0.000289 | 0.004107 |
| pag1 | 1.321635 | 1.136661 | 1.536711 | 0.000289 | 0.004107 |
| cd4 | 1.582008 | 1.233598 | 2.02882 | 0.000301 | 0.004257 |
| sema3f | 1.813553 | 1.312785 | 2.505342 | 0.000305 | 0.004292 |
| rab6a | 0.679536 | 0.550935 | 0.838154 | 0.000307 | 0.004292 |
| fabp4 | 1.286957 | 1.121934 | 1.476253 | 0.000314 | 0.004353 |
| ryr1 | 1.471229 | 1.192568 | 1.815002 | 0.000314 | 0.004353 |
| tnfrsf11a | 1.447561 | 1.183495 | 1.770545 | 0.000319 | 0.004397 |
| osm | 1.269891 | 1.114808 | 1.446548 | 0.000324 | 0.004445 |
| itgb5 | 0.617829 | 0.475121 | 0.8034 | 0.000326 | 0.004456 |
| defa1_defa1b | 1.310443 | 1.130551 | 1.51896 | 0.000332 | 0.004518 |
| tnfrsf4 | 1.476448 | 1.192615 | 1.82783 | 0.000347 | 0.004701 |
| cstb | 1.439789 | 1.178241 | 1.759396 | 0.000366 | 0.004928 |
| bpifb1 | 1.282549 | 1.118118 | 1.471161 | 0.000378 | 0.005071 |
| rbp5 | 1.308282 | 1.127845 | 1.517587 | 0.000387 | 0.005164 |
| retn | 1.416795 | 1.16828 | 1.718173 | 0.000399 | 0.005305 |
| ctsv | 0.650782 | 0.512859 | 0.825797 | 0.000408 | 0.005367 |
| ntrk3 | 0.47842 | 0.317939 | 0.719905 | 0.000406 | 0.005367 |
| nppb | 1.127038 | 1.054615 | 1.204434 | 0.000417 | 0.005463 |
| cd300lg | 0.635767 | 0.494183 | 0.817916 | 0.000426 | 0.005554 |
| lpa | 1.116299 | 1.049903 | 1.186893 | 0.000437 | 0.005656 |
| pilra | 1.469631 | 1.185861 | 1.821306 | 0.000436 | 0.005656 |
| commd9 | 0.560473 | 0.405681 | 0.774329 | 0.000447 | 0.005751 |
| dpp4 | 0.561094 | 0.40611 | 0.775227 | 0.000459 | 0.005885 |
| ada2 | 1.423886 | 1.168295 | 1.735393 | 0.000464 | 0.005893 |
| krt19 | 1.249166 | 1.10288 | 1.414855 | 0.000464 | 0.005893 |
| chrdl1 | 1.619052 | 1.235781 | 2.121193 | 0.000472 | 0.005977 |
| cdan1 | 1.865697 | 1.314294 | 2.648438 | 0.000485 | 0.00611 |
| cntn1 | 0.57103 | 0.416661 | 0.782591 | 0.000493 | 0.006186 |
| ca14 | 0.65037 | 0.510359 | 0.828792 | 0.000505 | 0.006306 |
| adam12 | 1.619441 | 1.233818 | 2.125588 | 0.000512 | 0.006374 |
| ccl23 | 1.461838 | 1.178673 | 1.813032 | 0.000547 | 0.006751 |
| pilrb | 1.345901 | 1.137295 | 1.592771 | 0.000546 | 0.006751 |
| cntn5 | 0.640697 | 0.497192 | 0.825621 | 0.00058 | 0.007117 |
| lilra5 | 1.61196 | 1.227173 | 2.117399 | 0.000601 | 0.007352 |
| fabp1 | 1.167147 | 1.068304 | 1.275135 | 0.000618 | 0.007501 |
| tgfa | 1.373411 | 1.145326 | 1.646919 | 0.000616 | 0.007501 |
| fap | 0.560253 | 0.401974 | 0.780857 | 0.000626 | 0.007556 |
| il16 | 1.3739 | 1.145146 | 1.648349 | 0.00063 | 0.007573 |
| pon1 | 0.544205 | 0.383791 | 0.771667 | 0.000639 | 0.00765 |
| cfc1 | 1.349601 | 1.13601 | 1.603351 | 0.000648 | 0.007719 |
| efemp1 | 1.598848 | 1.220859 | 2.093867 | 0.00065 | 0.007719 |
| dsc2 | 1.547651 | 1.203577 | 1.990088 | 0.000663 | 0.007849 |
| pamr1 | 1.789717 | 1.279615 | 2.503165 | 0.000673 | 0.007932 |
| msmb | 1.250182 | 1.098729 | 1.422512 | 0.000702 | 0.008202 |
| spon2 | 1.526571 | 1.195343 | 1.949583 | 0.000699 | 0.008202 |
| serpind1 | 1.870945 | 1.300417 | 2.691779 | 0.000737 | 0.008586 |
| hjv | 1.422245 | 1.158885 | 1.745455 | 0.000748 | 0.008677 |
| gast | 1.125744 | 1.050646 | 1.20621 | 0.000772 | 0.008887 |
| nectin2 | 1.597238 | 1.215773 | 2.098393 | 0.00077 | 0.008887 |
| col15a1 | 1.808307 | 1.279781 | 2.555105 | 0.000783 | 0.008981 |
| smad3 | 0.682697 | 0.546039 | 0.853557 | 0.00081 | 0.009247 |
| tgfbr2 | 1.467083 | 1.171999 | 1.836462 | 0.000822 | 0.009353 |
| sfrp1 | 1.380596 | 1.141508 | 1.669761 | 0.000887 | 0.010051 |
| clec14a | 1.56908 | 1.201958 | 2.048335 | 0.000924 | 0.01043 |
| itih4 | 1.952121 | 1.311164 | 2.906408 | 0.000987 | 0.011058 |
| tnfrsf17 | 1.501148 | 1.178875 | 1.911523 | 0.000986 | 0.011058 |
| pltp | 0.672888 | 0.531517 | 0.85186 | 0.000993 | 0.011082 |
| klk14 | 1.368689 | 1.135034 | 1.650445 | 0.001016 | 0.011197 |
| nos3 | 1.270424 | 1.10137 | 1.465426 | 0.001019 | 0.011197 |
| scrg1 | 1.671512 | 1.230406 | 2.270757 | 0.001015 | 0.011197 |
| serpina3 | 2.362274 | 1.414543 | 3.944978 | 0.001018 | 0.011197 |
| cd7 | 1.446753 | 1.160029 | 1.804345 | 0.001048 | 0.011477 |
| igfbp6 | 1.519481 | 1.181955 | 1.953392 | 0.001097 | 0.011941 |
| mad1l1 | 1.266588 | 1.099013 | 1.459713 | 0.001099 | 0.011941 |
| epha1 | 1.614919 | 1.21059 | 2.15429 | 0.001115 | 0.012072 |
| efna1 | 1.582404 | 1.200465 | 2.085861 | 0.001129 | 0.012173 |
| adamts15 | 1.412372 | 1.147128 | 1.738948 | 0.001141 | 0.012259 |
| ddc | 0.715519 | 0.584742 | 0.875546 | 0.001152 | 0.012331 |
| lilrb2 | 1.578204 | 1.197106 | 2.080624 | 0.001213 | 0.012941 |
| cd300a | 1.722515 | 1.237772 | 2.397097 | 0.001259 | 0.013359 |
| ncam1 | 0.624736 | 0.469374 | 0.831524 | 0.001261 | 0.013359 |
| cd5 | 1.417589 | 1.146434 | 1.752878 | 0.001275 | 0.013453 |
| rnase10 | 1.388502 | 1.136733 | 1.696035 | 0.001302 | 0.013693 |
| ahnak | 1.61629 | 1.203652 | 2.170388 | 0.001411 | 0.01478 |
| itga11 | 0.647198 | 0.495181 | 0.845882 | 0.001446 | 0.015044 |
| itgav | 0.42186 | 0.248068 | 0.717406 | 0.001443 | 0.015044 |
| bcan | 0.66184 | 0.513267 | 0.853421 | 0.001463 | 0.015164 |
| tgfbr1 | 1.766817 | 1.244057 | 2.509243 | 0.001472 | 0.015208 |
| lrig1 | 1.504759 | 1.169317 | 1.93643 | 0.001496 | 0.01534 |
| mmp9 | 1.279394 | 1.098908 | 1.489524 | 0.001495 | 0.01534 |
| cd38 | 1.52925 | 1.175386 | 1.989649 | 0.001559 | 0.015937 |
| a1bg | 2.913772 | 1.500474 | 5.658256 | 0.001587 | 0.016161 |
| ccn1 | 1.239089 | 1.083939 | 1.416447 | 0.001684 | 0.017059 |
| ptp4a3 | 1.326119 | 1.111935 | 1.58156 | 0.001687 | 0.017059 |
| serpina11 | 1.435936 | 1.145335 | 1.800269 | 0.001712 | 0.017253 |
| clgn | 1.383993 | 1.129517 | 1.6958 | 0.00172 | 0.017274 |
| rgma | 0.630801 | 0.472842 | 0.84153 | 0.001729 | 0.017309 |
| ccl21 | 1.371523 | 1.125437 | 1.671416 | 0.001741 | 0.017368 |
| ctss | 2.074269 | 1.312954 | 3.277031 | 0.001767 | 0.01754 |
| yap1 | 1.644588 | 1.20394 | 2.246517 | 0.00177 | 0.01754 |
| ermap | 1.448698 | 1.147369 | 1.829163 | 0.001837 | 0.01814 |
| cd274 | 1.47182 | 1.154016 | 1.877143 | 0.001845 | 0.018155 |
| scg2 | 1.52514 | 1.168846 | 1.99004 | 0.001876 | 0.018398 |
| nt5c1a | 1.497762 | 1.160685 | 1.93273 | 0.0019 | 0.018573 |
| pglyrp1 | 1.367903 | 1.122347 | 1.667183 | 0.001914 | 0.018644 |
| tnfrsf12a | 1.437912 | 1.142417 | 1.809838 | 0.001972 | 0.019154 |
| c1qa | 1.845645 | 1.251542 | 2.721768 | 0.001988 | 0.019203 |
| chi3l1 | 1.174007 | 1.06048 | 1.299687 | 0.001991 | 0.019203 |
| skap1 | 1.21287 | 1.073082 | 1.370868 | 0.002009 | 0.019316 |
| ccl27 | 1.272138 | 1.091194 | 1.483088 | 0.002106 | 0.02015 |
| polr2f | 1.374727 | 1.12228 | 1.68396 | 0.002109 | 0.02015 |
| clec6a | 1.344822 | 1.112704 | 1.625361 | 0.002179 | 0.020746 |
| adam8 | 1.554692 | 1.171676 | 2.062914 | 0.002229 | 0.021104 |
| wfikkn2 | 0.66567 | 0.512821 | 0.864075 | 0.002231 | 0.021104 |
| npdc1 | 1.452752 | 1.143008 | 1.846434 | 0.00227 | 0.021402 |
| prtn3 | 1.294031 | 1.096482 | 1.527173 | 0.002291 | 0.021528 |
| lgals3bp | 1.563235 | 1.172848 | 2.083563 | 0.002307 | 0.021615 |
| bambi | 1.556275 | 1.170708 | 2.068828 | 0.002327 | 0.021726 |
| clec12a | 2.010318 | 1.28204 | 3.1523 | 0.002346 | 0.021842 |
| ephb4 | 1.670843 | 1.199973 | 2.326483 | 0.002371 | 0.022002 |
| apoa1 | 0.506263 | 0.326227 | 0.785658 | 0.002399 | 0.022188 |
| nt5c3a | 0.847729 | 0.761847 | 0.943292 | 0.002436 | 0.022462 |
| ptprb | 1.710664 | 1.208197 | 2.422098 | 0.002479 | 0.022785 |
| btn3a2 | 1.417188 | 1.130475 | 1.776617 | 0.0025 | 0.022887 |
| tnfsf13 | 1.646375 | 1.191589 | 2.274736 | 0.002506 | 0.022887 |
| fas | 1.377255 | 1.118916 | 1.69524 | 0.002527 | 0.02301 |
| krt8 | 1.16583 | 1.055167 | 1.288098 | 0.002568 | 0.023268 |
| stc1 | 1.317815 | 1.101378 | 1.576785 | 0.002571 | 0.023268 |
| sema3g | 1.397401 | 1.124077 | 1.737183 | 0.002585 | 0.02332 |
| apcs | 1.687915 | 1.200558 | 2.373111 | 0.0026 | 0.023383 |
| hip1r | 1.30426 | 1.096932 | 1.550775 | 0.002635 | 0.023554 |
| spon1 | 1.463241 | 1.141798 | 1.875179 | 0.002632 | 0.023554 |
| anxa10 | 1.170987 | 1.056408 | 1.297993 | 0.002661 | 0.023711 |
| palm2 | 1.433828 | 1.133361 | 1.813952 | 0.00267 | 0.023724 |
| rida | 1.349896 | 1.109315 | 1.642653 | 0.002737 | 0.02424 |
| gas6 | 1.763088 | 1.215773 | 2.556793 | 0.002788 | 0.024617 |
| ccl11 | 1.451796 | 1.136887 | 1.853934 | 0.002805 | 0.024694 |
| vwa1 | 1.38118 | 1.117175 | 1.707573 | 0.002848 | 0.024997 |
| cxcl9 | 1.215554 | 1.069206 | 1.381934 | 0.002861 | 0.025004 |
| mxra8 | 0.591007 | 0.418271 | 0.835078 | 0.002866 | 0.025004 |
| lrrc25 | 1.368755 | 1.112371 | 1.684231 | 0.003014 | 0.02622 |
| cxcl13 | 1.199298 | 1.063317 | 1.352668 | 0.003078 | 0.026697 |
| adamtsl2 | 1.479468 | 1.140771 | 1.918723 | 0.003148 | 0.027226 |
| lrrc38 | 1.365171 | 1.109995 | 1.679008 | 0.003194 | 0.027541 |
| fgf21 | 1.111219 | 1.035682 | 1.192267 | 0.003324 | 0.028577 |
| efcab14 | 1.508466 | 1.146328 | 1.985007 | 0.003336 | 0.028592 |
| gpc5 | 0.776898 | 0.656273 | 0.919694 | 0.003364 | 0.028592 |
| idua | 1.343692 | 1.103026 | 1.636869 | 0.003349 | 0.028592 |
| scn4b | 1.439042 | 1.128276 | 1.835403 | 0.003365 | 0.028592 |
| pth | 1.206514 | 1.06414 | 1.367936 | 0.003386 | 0.028689 |
| clec10a | 1.46357 | 1.133844 | 1.889183 | 0.003451 | 0.029152 |
| nmnat1 | 1.168248 | 1.052483 | 1.296747 | 0.003492 | 0.029419 |
| adamts1 | 1.54889 | 1.154682 | 2.07768 | 0.003504 | 0.029427 |
| asgr2 | 1.75832 | 1.203539 | 2.568831 | 0.003525 | 0.029522 |
| gal | 0.814006 | 0.708869 | 0.934737 | 0.00354 | 0.029568 |
| ism1 | 1.394448 | 1.114866 | 1.744142 | 0.003587 | 0.02987 |
| pi3 | 1.256479 | 1.077469 | 1.46523 | 0.003598 | 0.029874 |
| b4gat1 | 0.568491 | 0.388515 | 0.831838 | 0.003638 | 0.030123 |
| bmper | 1.778951 | 1.205683 | 2.624793 | 0.003703 | 0.030573 |
| layn | 1.410035 | 1.117074 | 1.779827 | 0.003832 | 0.031553 |
| aplp1 | 0.764003 | 0.636505 | 0.917041 | 0.003857 | 0.031589 |
| vegfa | 1.252697 | 1.07517 | 1.459535 | 0.003858 | 0.031589 |
| prg2 | 1.337205 | 1.097823 | 1.628784 | 0.003885 | 0.031724 |
| itgam | 0.64386 | 0.476884 | 0.869302 | 0.004048 | 0.032907 |
| klrk1 | 1.365621 | 1.104181 | 1.688963 | 0.004053 | 0.032907 |
| ment | 0.525821 | 0.338878 | 0.81589 | 0.004134 | 0.033474 |
| ctag1a_ctag1b | 1.332987 | 1.094937 | 1.622791 | 0.004189 | 0.033823 |
| itgb1 | 0.512188 | 0.323801 | 0.810179 | 0.004241 | 0.034075 |
| notch2 | 0.455447 | 0.265657 | 0.780825 | 0.004243 | 0.034075 |
| ltbp2 | 1.483827 | 1.131341 | 1.946136 | 0.004348 | 0.034821 |
| hdac9 | 1.195339 | 1.056898 | 1.351914 | 0.004496 | 0.035905 |
| lect2 | 1.25129 | 1.071762 | 1.460891 | 0.004553 | 0.036266 |
| mcam | 0.658996 | 0.493202 | 0.880524 | 0.004795 | 0.038086 |
| adamts8 | 0.748104 | 0.611291 | 0.915538 | 0.004858 | 0.038483 |
| ptprn2 | 1.446682 | 1.118536 | 1.871095 | 0.004902 | 0.038722 |
| ccl3 | 1.190657 | 1.05424 | 1.344725 | 0.004943 | 0.038944 |
| tfrc | 1.383167 | 1.1021 | 1.735914 | 0.00513 | 0.040307 |
| ceacam8 | 1.28548 | 1.077694 | 1.53333 | 0.005242 | 0.041082 |
| vstm2l | 1.217703 | 1.06038 | 1.398367 | 0.005261 | 0.041119 |
| cd72 | 1.376514 | 1.0993 | 1.723635 | 0.005351 | 0.04171 |
| fcamr | 1.218267 | 1.059896 | 1.400301 | 0.005458 | 0.042317 |
| pnliprp1 | 1.199089 | 1.054963 | 1.362905 | 0.005455 | 0.042317 |
| ccl15 | 1.318445 | 1.08468 | 1.60259 | 0.005499 | 0.042409 |
| osmr | 1.93854 | 1.21489 | 3.093234 | 0.005496 | 0.042409 |
| saa4 | 1.383035 | 1.099576 | 1.739567 | 0.005586 | 0.042968 |
| boc | 0.565935 | 0.377879 | 0.847578 | 0.005737 | 0.044014 |
| c1s | 2.014751 | 1.22548 | 3.312353 | 0.005753 | 0.044017 |
| tab2 | 0.862929 | 0.77712 | 0.958213 | 0.005803 | 0.044285 |
| aspscr1 | 0.792001 | 0.671037 | 0.934771 | 0.005822 | 0.044316 |
| csnk2a1 | 0.704822 | 0.549603 | 0.903878 | 0.005847 | 0.04439 |
| cyp24a1 | 1.162582 | 1.044225 | 1.294355 | 0.005961 | 0.045138 |
| cd34 | 0.583157 | 0.396608 | 0.85745 | 0.006109 | 0.046143 |
| kitlg | 0.719647 | 0.568745 | 0.910586 | 0.006142 | 0.046274 |
| umod | 0.797857 | 0.678757 | 0.937856 | 0.006185 | 0.046477 |
| ctsz | 1.472329 | 1.114954 | 1.944254 | 0.006391 | 0.047775 |
| nell1 | 0.739569 | 0.59541 | 0.918632 | 0.006388 | 0.047775 |
| mamdc2 | 1.465581 | 1.112566 | 1.930605 | 0.006556 | 0.048884 |
| mpo | 1.285083 | 1.072422 | 1.539914 | 0.006578 | 0.048924 |
| c1r | 1.810396 | 1.179078 | 2.779742 | 0.00667 | 0.049473 |
| slc4a1 | 0.722351 | 0.571044 | 0.913749 | 0.006686 | 0.049473 |

# Supplementary table 13. Aassociation between proteins and AA in women.

| **Protein** | **HR** | **95%LCI** | **95%UCI** | **P-Value** | **pBH** |
| --- | --- | --- | --- | --- | --- |
| mmp12 | 3.223486 | 2.488432 | 4.175666 | 7.73E-19 | 2.26E-15 |
| cxcl17 | 2.552609 | 2.010422 | 3.241016 | 1.44E-14 | 2.11E-11 |
| wfdc2 | 2.841548 | 2.141475 | 3.770484 | 4.60E-13 | 4.48E-10 |
| tnfrsf10b | 1.630641 | 1.425709 | 1.86503 | 9.62E-13 | 7.03E-10 |
| plaur | 5.865079 | 3.588635 | 9.585582 | 1.69E-12 | 9.87E-10 |
| lamp3 | 2.412682 | 1.802467 | 3.229482 | 3.22E-09 | 1.35E-06 |
| gdf15 | 2.132278 | 1.659397 | 2.739916 | 3.24E-09 | 1.35E-06 |
| pgf | 3.027618 | 2.09442 | 4.376614 | 3.82E-09 | 1.39E-06 |
| alpp | 1.431994 | 1.269622 | 1.615131 | 4.98E-09 | 1.62E-06 |
| tnfsf13b | 2.71071 | 1.938201 | 3.791117 | 5.66E-09 | 1.65E-06 |
| igfbp4 | 2.282017 | 1.724668 | 3.019481 | 7.70E-09 | 2.05E-06 |
| acvrl1 | 4.471132 | 2.65539 | 7.52847 | 1.77E-08 | 4.30E-06 |
| pigr | 2.948907 | 1.98995 | 4.369985 | 7.09E-08 | 1.59E-05 |
| sftpa2 | 1.918885 | 1.509293 | 2.439631 | 1.04E-07 | 2.16E-05 |
| il19 | 1.754208 | 1.423957 | 2.161053 | 1.28E-07 | 2.36E-05 |
| il6 | 1.458804 | 1.268013 | 1.678302 | 1.29E-07 | 2.36E-05 |
| tnfrsf10a | 2.308512 | 1.690591 | 3.152287 | 1.41E-07 | 2.43E-05 |
| agrn | 2.763734 | 1.890596 | 4.040116 | 1.54E-07 | 2.50E-05 |
| msln | 1.938375 | 1.512785 | 2.483696 | 1.67E-07 | 2.57E-05 |
| ntprobnp | 1.49021 | 1.28215 | 1.732033 | 2.00E-07 | 2.92E-05 |
| eda2r | 2.633485 | 1.820445 | 3.809641 | 2.75E-07 | 3.82E-05 |
| clec5a | 3.333857 | 2.096569 | 5.301329 | 3.61E-07 | 4.80E-05 |
| prss8 | 2.595914 | 1.79638 | 3.751305 | 3.81E-07 | 4.84E-05 |
| cst3 | 3.358055 | 2.09312 | 5.387429 | 5.10E-07 | 6.21E-05 |
| tnfrsf1a | 3.027508 | 1.943673 | 4.715713 | 9.62E-07 | 0.000112 |
| efna4 | 2.665486 | 1.771409 | 4.010828 | 2.57E-06 | 0.000289 |
| il15 | 2.646915 | 1.759673 | 3.981512 | 2.97E-06 | 0.000321 |
| cd83 | 2.691401 | 1.772515 | 4.086643 | 3.38E-06 | 0.000341 |
| col6a3 | 2.434844 | 1.673353 | 3.542868 | 3.31E-06 | 0.000341 |
| klk13 | 2.37322 | 1.639958 | 3.434338 | 4.58E-06 | 0.000446 |
| havcr2 | 2.625607 | 1.725939 | 3.99424 | 6.49E-06 | 0.000593 |
| vsig4 | 2.202772 | 1.563437 | 3.103551 | 6.34E-06 | 0.000593 |
| lrg1 | 3.906229 | 2.152702 | 7.088126 | 7.39E-06 | 0.000655 |
| tnfrsf1b | 1.715816 | 1.353216 | 2.175578 | 8.30E-06 | 0.000714 |
| rnase1 | 2.467927 | 1.656023 | 3.677886 | 9.08E-06 | 0.000734 |
| ccl3 | 1.404258 | 1.20858 | 1.631617 | 9.24E-06 | 0.000734 |
| rab6a | 0.42539 | 0.291519 | 0.620737 | 9.29E-06 | 0.000734 |
| lilrb4 | 2.219556 | 1.557248 | 3.163547 | 1.04E-05 | 0.000796 |
| scarb2 | 2.566581 | 1.677933 | 3.925867 | 1.38E-05 | 0.001035 |
| spink1 | 2.008006 | 1.463126 | 2.755803 | 1.59E-05 | 0.001159 |
| lgals4 | 1.869385 | 1.406466 | 2.484666 | 1.64E-05 | 0.001166 |
| colec12 | 3.327862 | 1.921862 | 5.762467 | 1.77E-05 | 0.001199 |
| tnfrsf11a | 2.182704 | 1.528296 | 3.117326 | 1.77E-05 | 0.001199 |
| tgfa | 1.946948 | 1.435779 | 2.640104 | 1.81E-05 | 0.001199 |
| tnfrsf6b | 1.688611 | 1.325403 | 2.15135 | 2.24E-05 | 0.001453 |
| serpina3 | 7.991301 | 3.032134 | 21.06136 | 2.63E-05 | 0.00167 |
| adm | 3.617732 | 1.982853 | 6.600582 | 2.77E-05 | 0.001725 |
| tafa5 | 2.407477 | 1.593339 | 3.637611 | 3.02E-05 | 0.001807 |
| fgf23 | 1.573204 | 1.271507 | 1.946486 | 3.03E-05 | 0.001807 |
| ltbr | 2.989329 | 1.782293 | 5.013816 | 3.32E-05 | 0.001941 |
| il4r | 2.178106 | 1.50383 | 3.154708 | 3.81E-05 | 0.002183 |
| pik3ip1 | 2.97795 | 1.770545 | 5.008731 | 3.90E-05 | 0.00219 |
| rbp2 | 1.571922 | 1.266237 | 1.951403 | 4.14E-05 | 0.002284 |
| msr1 | 2.222299 | 1.514006 | 3.26195 | 4.54E-05 | 0.002457 |
| orm1 | 5.911631 | 2.506302 | 13.9438 | 4.94E-05 | 0.002604 |
| cdcp1 | 1.926702 | 1.403415 | 2.645106 | 4.99E-05 | 0.002604 |
| spp1 | 2.073055 | 1.456304 | 2.951005 | 5.20E-05 | 0.002667 |
| ckap4 | 2.737151 | 1.677989 | 4.464864 | 5.50E-05 | 0.002772 |
| vwc2 | 2.424495 | 1.571484 | 3.740525 | 6.25E-05 | 0.003095 |
| nectin2 | 2.881402 | 1.711668 | 4.85052 | 6.82E-05 | 0.003178 |
| lgals9 | 2.560673 | 1.611863 | 4.067991 | 6.85E-05 | 0.003178 |
| tff2 | 1.720676 | 1.318232 | 2.245983 | 6.54E-05 | 0.003178 |
| fabp1 | 1.398731 | 1.185798 | 1.6499 | 6.82E-05 | 0.003178 |
| col18a1 | 4.212982 | 2.069194 | 8.577842 | 7.36E-05 | 0.003307 |
| igfbp7 | 2.218462 | 1.496651 | 3.28839 | 7.25E-05 | 0.003307 |
| nppb | 1.333569 | 1.15643 | 1.53784 | 7.54E-05 | 0.003337 |
| icam5 | 2.207281 | 1.486295 | 3.278009 | 8.71E-05 | 0.003799 |
| relt | 2.779949 | 1.660057 | 4.65533 | 0.000102 | 0.004366 |
| cd74 | 2.445937 | 1.556136 | 3.844527 | 0.000106 | 0.004487 |
| fstl3 | 2.341867 | 1.52203 | 3.603308 | 0.000109 | 0.004533 |
| efna1 | 2.795504 | 1.651689 | 4.731427 | 0.000129 | 0.005244 |
| il15ra | 2.638088 | 1.605398 | 4.335066 | 0.000129 | 0.005244 |
| crhbp | 3.36353 | 1.805078 | 6.267504 | 0.000134 | 0.005345 |
| siglec1 | 2.053261 | 1.41875 | 2.971546 | 0.000136 | 0.005387 |
| clec10a | 2.622321 | 1.59641 | 4.307521 | 0.000141 | 0.005476 |
| cd27 | 2.039398 | 1.411446 | 2.946724 | 0.000148 | 0.005672 |
| epha2 | 2.10265 | 1.425593 | 3.101262 | 0.000178 | 0.006669 |
| pi3 | 1.641951 | 1.267169 | 2.127579 | 0.000176 | 0.006669 |
| ccl7 | 1.480158 | 1.204986 | 1.818169 | 0.000186 | 0.006892 |
| inhbb | 1.980085 | 1.381972 | 2.837061 | 0.000197 | 0.007188 |
| csf1 | 2.874022 | 1.64282 | 5.027941 | 0.000216 | 0.007425 |
| il2ra | 2.087206 | 1.413397 | 3.08224 | 0.000216 | 0.007425 |
| cgref1 | 2.082667 | 1.412064 | 3.071747 | 0.000215 | 0.007425 |
| rnase6 | 1.986947 | 1.381311 | 2.858126 | 0.000214 | 0.007425 |
| trem2 | 1.82682 | 1.328772 | 2.511547 | 0.000207 | 0.007425 |
| clec14a | 2.55375 | 1.55246 | 4.200841 | 0.000222 | 0.00756 |
| areg | 1.779172 | 1.308749 | 2.418686 | 0.000236 | 0.007915 |
| tnr | 0.47306 | 0.316572 | 0.706904 | 0.00026 | 0.008624 |
| ccn3 | 2.178137 | 1.431967 | 3.31312 | 0.000275 | 0.008927 |
| reg4 | 1.817827 | 1.317646 | 2.507878 | 0.000273 | 0.008927 |
| il18bp | 2.432307 | 1.499957 | 3.944191 | 0.000314 | 0.010069 |
| ephb4 | 2.822208 | 1.602684 | 4.969698 | 0.000326 | 0.010249 |
| krt19 | 1.495596 | 1.20059 | 1.863089 | 0.00033 | 0.010249 |
| gal | 0.581814 | 0.432972 | 0.781824 | 0.000327 | 0.010249 |
| defb4a_defb4b | 1.232709 | 1.099109 | 1.382549 | 0.000351 | 0.01079 |
| spon1 | 2.088228 | 1.393948 | 3.128305 | 0.000356 | 0.010841 |
| tnfrsf4 | 1.998106 | 1.365362 | 2.924081 | 0.000367 | 0.011048 |
| mzb1 | 1.716165 | 1.273423 | 2.312838 | 0.000389 | 0.011589 |
| dsc2 | 2.01875 | 1.366268 | 2.982835 | 0.000421 | 0.012416 |
| asgr1 | 2.503406 | 1.500263 | 4.177296 | 0.000444 | 0.012854 |
| il10rb | 2.35694 | 1.460267 | 3.804212 | 0.000448 | 0.012854 |
| klk10 | 1.796299 | 1.295153 | 2.491359 | 0.000449 | 0.012854 |
| cd4 | 1.823092 | 1.303059 | 2.550663 | 0.000457 | 0.012957 |
| cbln4 | 0.384809 | 0.225297 | 0.657257 | 0.000471 | 0.013244 |
| tnfsf13 | 3.123855 | 1.645625 | 5.929948 | 0.000496 | 0.013659 |
| icam1 | 2.544479 | 1.504964 | 4.302012 | 0.000491 | 0.013659 |
| tnfrsf12a | 2.184244 | 1.405255 | 3.395057 | 0.000517 | 0.014116 |
| ambp | 4.079819 | 1.84251 | 9.033828 | 0.000527 | 0.014252 |
| npdc1 | 2.159444 | 1.396823 | 3.338431 | 0.000533 | 0.014293 |
| enpp5 | 0.598204 | 0.44717 | 0.80025 | 0.000539 | 0.014305 |
| shisa5 | 2.230972 | 1.414254 | 3.519339 | 0.00056 | 0.014743 |
| nectin4 | 2.157078 | 1.390399 | 3.346512 | 0.000602 | 0.015694 |
| ccl22 | 1.655485 | 1.240211 | 2.209809 | 0.000624 | 0.016138 |
| fabp2 | 1.439857 | 1.167867 | 1.775193 | 0.000643 | 0.016489 |
| fgf5 | 2.105996 | 1.369788 | 3.237886 | 0.000689 | 0.017485 |
| capg | 1.60892 | 1.222214 | 2.117978 | 0.000697 | 0.017485 |
| ret | 0.472629 | 0.306419 | 0.728998 | 0.0007 | 0.017485 |
| lrrn1 | 0.451004 | 0.28418 | 0.715762 | 0.000727 | 0.018013 |
| chchd10 | 2.002741 | 1.337564 | 2.998713 | 0.000746 | 0.018313 |
| fas | 1.701217 | 1.248737 | 2.317652 | 0.000757 | 0.018439 |
| cd300e | 1.887686 | 1.303533 | 2.733617 | 0.000771 | 0.018612 |
| angpt2 | 2.084663 | 1.357118 | 3.202241 | 0.000796 | 0.019058 |
| spon2 | 2.18404 | 1.381339 | 3.453192 | 0.000832 | 0.019755 |
| ca12 | 2.136356 | 1.365046 | 3.34349 | 0.000895 | 0.020919 |
| ncan | 0.465895 | 0.296936 | 0.730994 | 0.000889 | 0.020919 |
| serping1 | 6.563844 | 2.123627 | 20.28796 | 0.001083 | 0.024916 |
| hspg2 | 2.354662 | 1.409042 | 3.934898 | 0.00108 | 0.024916 |
| tnfrsf21 | 2.837921 | 1.516872 | 5.309478 | 0.0011 | 0.025113 |
| hla_e | 2.341042 | 1.402002 | 3.909034 | 0.001147 | 0.025983 |
| timd4 | 1.870117 | 1.281347 | 2.729423 | 0.001174 | 0.02639 |
| ccl18 | 1.379509 | 1.134985 | 1.676713 | 0.00123 | 0.027426 |
| ceacam6 | 2.009769 | 1.314635 | 3.072466 | 0.001268 | 0.028068 |
| sel1l | 1.800357 | 1.257801 | 2.576947 | 0.001311 | 0.028808 |
| fap | 0.349882 | 0.184213 | 0.664541 | 0.001334 | 0.029093 |
| dpp6 | 0.427631 | 0.25442 | 0.718765 | 0.001344 | 0.029096 |
| ceacam5 | 1.437189 | 1.150415 | 1.79545 | 0.001404 | 0.030157 |
| bpifb1 | 1.600192 | 1.198564 | 2.136402 | 0.001431 | 0.03052 |
| oscar | 2.341798 | 1.383397 | 3.96417 | 0.001533 | 0.032456 |
| nbl1 | 2.614972 | 1.432061 | 4.774993 | 0.001755 | 0.036624 |
| cstb | 1.8361 | 1.255057 | 2.686144 | 0.001746 | 0.036624 |
| efemp1 | 2.28643 | 1.361514 | 3.839668 | 0.001768 | 0.036632 |
| rnf149 | 2.133683 | 1.324899 | 3.436189 | 0.001826 | 0.037315 |
| ifi30 | 2.110384 | 1.319585 | 3.375094 | 0.001824 | 0.037315 |
| chi3l1 | 1.386533 | 1.128659 | 1.703326 | 0.001854 | 0.037611 |
| fam3c | 2.404252 | 1.382216 | 4.181999 | 0.001896 | 0.038209 |
| ptgds | 2.147858 | 1.324093 | 3.484119 | 0.001952 | 0.039075 |
| olr1 | 1.471268 | 1.151675 | 1.879548 | 0.002001 | 0.039771 |
| clec4d | 1.523083 | 1.165427 | 1.990499 | 0.002063 | 0.040457 |
| b4gat1 | 0.313671 | 0.150046 | 0.655731 | 0.002059 | 0.040457 |
| dll1 | 2.311434 | 1.351761 | 3.952419 | 0.002205 | 0.042949 |
| cd300lf | 1.703446 | 1.210597 | 2.39694 | 0.002238 | 0.043307 |
| hyou1 | 3.417617 | 1.551261 | 7.529424 | 0.002293 | 0.043914 |
| timp1 | 2.254057 | 1.336265 | 3.802218 | 0.002314 | 0.043914 |
| tmed4 | 1.436171 | 1.137929 | 1.812581 | 0.002304 | 0.043914 |
| b2m | 1.587913 | 1.179069 | 2.138524 | 0.002331 | 0.04394 |
| qpct | 2.42486 | 1.369116 | 4.294705 | 0.002388 | 0.044443 |
| cst7 | 1.24335 | 1.080348 | 1.430946 | 0.002383 | 0.044443 |
| ca4 | 2.983188 | 1.470483 | 6.052031 | 0.002459 | 0.045482 |
| guca2a | 2.139992 | 1.306221 | 3.505964 | 0.002523 | 0.045862 |
| tnfrsf9 | 1.669113 | 1.197011 | 2.327413 | 0.002527 | 0.045862 |
| prss2 | 1.590135 | 1.177235 | 2.147855 | 0.002497 | 0.045862 |
| pglyrp1 | 1.732829 | 1.212274 | 2.476911 | 0.002561 | 0.046193 |
| btn2a1 | 2.485174 | 1.373123 | 4.497839 | 0.002634 | 0.046361 |
| cd79b | 1.678798 | 1.19832 | 2.35193 | 0.002598 | 0.046361 |
| vmo1 | 1.565079 | 1.168853 | 2.09562 | 0.002634 | 0.046361 |
| igdcc4 | 0.307786 | 0.142875 | 0.663045 | 0.002618 | 0.046361 |

# Supplementary table 14. Enrichment analysis results for Reactome pathways in men.

| **Reactome** | **Adjusted-p-value** | **Protein** |
| --- | --- | --- |
| Post-translational protein phosphorylation | 3.26E-14 | IL6, CST3, MSLN, SPP1, IGFBP4, SHISA5, IGFBP7, FAM20A, CKAP4, FGF23, SERPINA1, CSF1, TIMP1, FSTL3, CDH2, PENK, FGA, CHRDL1, SERPIND1, CCN1, SCG2, APOA1, GAS6, VWA1, MXRA8 |
| Regulation of Insulin-like Growth Factor (IGF) transport and uptake by Insulin-like Growth Factor Binding Proteins (IGFBPs) | 1.32E-13 | IL6, CST3, MSLN, SPP1, IGFBP4, SHISA5, IGFBP7, FAM20A, CKAP4, FGF23, SERPINA1, CSF1, TIMP1, FSTL3, CDH2, PENK, FGA, CHRDL1, SERPIND1, IGFBP6, CCN1, SCG2, APOA1, GAS6, VWA1, MXRA8 |
| Neutrophil degranulation | 2.08E-13 | NPC2, PLAUR, CST3, ORM1, GGH, S100A12, B2M, TNFRSF1B, LAIR1, CEACAM6, CLEC5A, RNASET2, PIGR, CLEC4D, CKAP4, PSAP, GM2A, SERPINA1, LRG1, CTSD, CFD, PRSS2, LCN2, OSCAR, LTA4H, RAB6A, CSTB, RETN, COMMD9, ADA2, SERPINA3, LILRB2, CD300A, ITGAV, MMP9, A1BG, CTSS, PGLYRP1, CHI3L1, ADAM8, PRTN3, CLEC12A, PTPRB, PRG2, ITGAM, PTPRN2, CEACAM8, CTSZ, MPO |
| TNFs bind their physiological receptors | 2.54E-12 | TNFRSF9, TNFRSF1B, TNFRSF1A, TNFRSF11B, TNFRSF6B, CD27, EDA2R, TNFRSF13B, TNFSF13B, EDAR, CD70, TNFRSF4, TNFRSF17, TNFSF13 |
| Immunoregulatory interactions between a Lymphoid and a non-Lymphoid cell | 1.13E-08 | TREM2, B2M, LILRB4, LAIR1, COLEC12, CD300LF, SIGLEC7, ICAM1, SIGLEC1, SIGLEC8, SFTPD, OSCAR, ICAM5, CD300E, IGLC2, CD300LG, PILRA, PILRB, LILRA5, NECTIN2, LILRB2, CD300A, NPDC1, KLRK1, ITGB1 |
| Degradation of the extracellular matrix | 9.03E-08 | MMP12, SPP1, COL18A1, CTSL, COL6A3, MMP7, FURIN, BSG, CTSD, PRSS2, TIMP1, HSPG2, ELN, CTSV, COL15A1, BCAN, MMP9, CTSS, ADAM8, ADAMTS1, ADAMTS8 |
| Integrin cell surface interactions | 2.69E-05 | SPP1, COL18A1, COL6A3, BSG, ICAM1, AGRN, HSPG2, ICAM5, FGA, ITGB5, ITGA11, ITGAV, ITGAM, ITGB1 |
| Signaling by Interleukins | 0.000125243 | IL6, HGF, S100A12, IL2RA, IL7R, LGALS9, TNFRSF1B, TNFRSF1A, HAVCR2, CD80, TNF, IL10RB, CSF1, IL18BP, ICAM1, CCL22, IL1RN, TIMP1, IL22, LCN2, IL18R1, CD4, OSM, IL16, SMAD3, MMP9, PRTN3, CCL11, VEGFA, ITGAM, ITGB1, CCL3, OSMR, TAB2 |
| Platelet degranulation | 0.000152274 | HGF, ORM1, RARRES2, PSAP, SERPINA1, CFD, ITIH3, TIMP1, FGA, ITIH4, SERPINA3, A1BG, LGALS3BP, APOA1, GAS6, VEGFA |
| Chemokine receptors bind chemokines | 0.000158639 | CCL7, CCL16, XCL1, CXCL16, CCL22, CCL21, CCL27, CCL11, CXCL9, CXCL13, CCL3 |
| Attachment and Entry | 0.004007336 | CTSL, FURIN, HAVCR1, AGRN, HSPG2, GPC5 |
| Peptide ligand-binding receptors | 0.012078613 | CCL7, CCL16, EDN1, XCL1, CXCL16, GPR37, MLN, PSAP, CCL22, PENK, CCL21, CCL27, CCL11, CXCL9, CXCL13, GAL, CCL3 |
| Signaling by TGFB family members | 0.013843809 | ACVRL1, INHBB, FURIN, FSTL3, ITGB5, CHRDL1, SMAD3, TGFBR2, ITGAV, TGFBR1, BAMBI, ITGB1, LTBP2 |
| Diseases of glycosylation | 0.014034112 | AGRN, HSPG2, OGN, NCAN, SPON2, ADAMTS15, BCAN, SPON1, ADAMTSL2, GPC5, ADAMTS1, B4GAT1, NOTCH2, ADAMTS8 |

IGF: insulin-like growth factor; IGFBP: insulin-like growth factor binding protein; TNF: tumor necrosis factor; TGFB: Transforming Growth Factor-Beta.

# Supplementary table 15. Enrichment analysis results for Reactome pathways in women.

| **Reactome** | **Adjusted-p-value** | **Protein** |
| --- | --- | --- |
| TNFs bind their physiological receptors | 1.05E-08 | TNFSF13B, EDA2R, TNFRSF1A, TNFRSF1B, TNFRSF6B, CD27, TNFRSF4, TNFSF13, TNFRSF9 |
| Regulation of Insulin-like Growth Factor (IGF) transport and uptake by Insulin-like Growth Factor Binding Proteins (IGFBPs) | 7.73E-07 | IGFBP4, IL6, MSLN, CST3, KLK13, FGF23, SPP1, CKAP4, IGFBP7, FSTL3, CSF1, SHISA5, TIMP1 |
| Post-translational protein phosphorylation | 1.54513E-06 | IGFBP4, IL6, MSLN, CST3, FGF23, SPP1, CKAP4, IGFBP7, FSTL3, CSF1, SHISA5, TIMP1 |
| Neutrophil degranulation | 0.000240061 | PLAUR, PIGR, CLEC5A, CST3, LRG1, TNFRSF1B, RAB6A, SERPINA3, ORM1, CKAP4, CEACAM6, OSCAR, CSTB, CHI3L1, OLR1, CLEC4D, B2M, QPCT, PRSS2, PGLYRP1 |
| Immunoregulatory interactions between a Lymphoid and a non-Lymphoid cell | 0.000568392 | LILRB4, COLEC12, NECTIN2, ICAM5, SIGLEC1, TREM2, ICAM1, NPDC1, CD300E, OSCAR, CD300LF, B2M |
| Extracellular matrix organization | 0.003981082 | MMP12, AGRN, COL6A3, SPP1, COL18A1, ICAM5, TNR, ICAM1, NCAN, HSPG2, CEACAM6, EFEMP1, TIMP1, PRSS2 |
| Signaling by Interleukins | 0.004093833 | IL19, IL6, TNFRSF1A, IL15, HAVCR2, TNFRSF1B, CCL3, IL4R, LGALS9, IL15RA, CSF1, IL2RA, IL18BP, IL10RB, CD4, ICAM1, CCL22, TIMP1 |
| Integrin cell surface interactions | 0.022960141 | AGRN, COL6A3, SPP1, COL18A1, ICAM5, ICAM1, HSPG2 |

IGF: insulin-like growth factor; IGFBP: insulin-like growth factor binding protein; TNF: tumor necrosis factor; TGFB: Transforming Growth Factor-Beta.

# Supplementary table 16. Association between Protein Risk Score and Risk of Aortic Aneurysm Using Fine-Gray Competing Risk Models with All-Cause Mortality as the Competing Event.

|  | **Adjust variables** | **HR per SD Protein Risk Score in men (95% CI)** | **HR per SD Protein Risk Score in women (95% CI)** |
| --- | --- | --- | --- |
| Internal validation set | Age and Clinical risks factors | 1.85 (1.44, 2.36) | 1.47 (1.08, 2.13) |
| Independent validation cohort | Age and Clinical risks factors | 2.27 (1.60, 3.20) | 2.30 (1.46, 3.82) |

# Supplementary table 17. Calibration Performance of the Predictive Models in the Independent Validation Cohort.

|  | **Calibration Slope (Clinical Risk Model)** | **Calibration Slope (Clinical Risk Model + Protein Risk Score)** |
| --- | --- | --- |
| **Male** | 0.8455 | 0.8891 |
| **Female** | 0.8164 | 0.8481 |

# Supplementary table 18. Predictive performance of the final model incorporating the protein risk score derived from LASSO regression following KNN imputation of missing protein measurements in internal and external validation cohorts.

| **Model** | **C statistic (men)** | **C statistic (women)** |
| --- | --- | --- |
| **Internal validation set** | | |
| Age + Clinical risk factors + protein risk score | 0.789 (0.721-0.858) | 0.829 (0.769-0.890) |
| **Independent validation cohort** | | |
| Age + Clinical risk factors + protein risk score | 0.810 (0.803-0.867) | 0.822 (0.764-0.850) |
